# Analysis of immune characteristics and inflammatory mechanisms in COPD patients: a multi-layered study combining bulk and single-cell transcriptome analysis and machine learning

**DOI:** 10.3389/fmed.2025.1592802

**Published:** 2025-07-21

**Authors:** Changjin Wei, Yongfeng Zhu, Caiming Chen, Feipeng Li, Li Zheng

**Affiliations:** Department of Respiratory Medicine, Jiangbin Hospital of Guangxi Zhuang Autonomous Region, Nanning, Guangxi, China

**Keywords:** COPD, inflammatory genes, immune microenvironment, risk prediction model, molecular docking

## Abstract

**Objective:**

This study aims to investigate the potential roles and mechanisms of inflammatory genes in COPD.

**Methods:**

Transcriptome data from the airway epithelial tissues of COPD patients and normal individuals were downloaded from the GEO database. Differential gene expression analysis was performed using R software and its limma package, followed by GO, KEGG, and GSEA enrichment analyses. Inflammatory-related differentially expressed genes were screened based on literature data and analyzed for pathway enrichment using the Metascape database. Inflammatory-related COPD feature genes were selected using Lasso regression and random forest algorithms, and a COPD risk prediction model was constructed. Differences between the immune microenvironment of COPD and normal samples were analyzed using the ESTIMATE algorithm, the CIBERSORT method, and single-cell sequencing data. COPD patients were clustered using the ConsensusClusterPlus algorithm, and the pathway activity differences of different inflammatory subtypes were analyzed using GSVA. Potential traditional Chinese medicine monomer components capable of targeting key biomarker proteins were screened using the HERB database, and their binding potential was evaluated through molecular docking and molecular dynamics simulations.

**Results:**

A total of 495 significantly differentially expressed genes were identified, showing distinct expression patterns between COPD patients and healthy individuals. Functional and pathway enrichment analyses revealed significant enrichment of processes such as keratinocyte differentiation, arachidonic acid metabolism, IL-17 signaling pathway, and TNF signaling pathway in COPD. Fourteen inflammatory-related COPD genes were identified, which were significantly enriched in immune system processes and inflammatory responses. Using Lasso regression and random forest algorithms, seven feature genes were selected to construct a COPD risk prediction model, which demonstrated good accuracy. Immune cell infiltration analysis revealed a significant increase in monocytes, M0 macrophages, and eosinophils in COPD patients. Clustering analysis identified two inflammatory subtypes, with genes such as CLEC5A and CXCL8 significantly upregulated in the C2 subtype. Cinnamaldehyde, a potential traditional Chinese medicine monomer component, was identified to potentially exert anti-inflammatory effects by targeting the CXCL8 protein.

**Conclusion:**

This study reveals significantly enriched biological processes and pathways in COPD patients, identifies multiple inflammatory-related COPD feature genes, and finds that cinnamaldehyde may have potential therapeutic effects on inflammatory subtypes of COPD.

## 1 Introduction

Chronic obstructive pulmonary disease (COPD) is a chronic inflammatory lung disease characterized by irreversible airflow limitation, usually caused by long-term exposure to harmful particles or gases (1, 2). COPD is currently the third leading cause of death globally (1, 3). Particularly among the elderly, the incidence and mortality rates of COPD have significantly increased, placing a substantial economic burden on patients and healthcare systems (4, 5). The pathogenesis of COPD is complex and diverse, including persistent inflammatory responses, airway remodeling, and alveolar destruction (4, 6). Despite some progress in understanding the pathophysiology of COPD, current treatments focus mainly on symptom management and prevention of acute exacerbations, lacking effective interventions to reverse or significantly slow disease progression (4, 7). Common treatments include bronchodilators, inhaled corticosteroids, and phosphodiesterase-4 inhibitors, but these drugs relieve symptoms only temporarily and cannot stop disease progression (7). Therefore, there is an urgent need to develop new treatment strategies to improve the prognosis and quality of life of COPD patients.

In recent years, the role of inflammation in the development and progression of COPD has received widespread attention. Studies have shown significant infiltration of inflammatory cells, including neutrophils, macrophages, and T lymphocytes, in the airways and lung tissues of COPD patients (8, 9). These inflammatory cells release various cytokines, chemokines, and proteases, leading to airway and alveolar structure destruction, subsequently causing airway remodeling and lung function decline (10–12). Neutrophils and macrophages play particularly prominent roles in COPD by secreting reactive oxygen species and proteases, directly causing tissue damage and inflammation (13–17). Additionally, certain inflammatory mediators, such as IL-17, TNF-*α*, and CXCL8, are significantly increased in COPD patients, participating in inflammatory responses and tissue destruction through various signaling pathways (18–22). These inflammatory processes not only exacerbate disease progression but are also closely related to comorbidities and disease complexity in elderly patients.

Several studies have employed bioinformatics frameworks to investigate the molecular characteristics of COPD. For example, Yu et al. conducted an integrated analysis of multiple COPD gene expression datasets and identified key genes using weighted gene co-expression network analysis (WGCNA) and Lasso regression, followed by immune infiltration profiling (23). Li et al. systematically screened potential diagnostic markers for COPD by integrating differential expression analysis, WGCNA, and three machine learning algorithms (24). Luo et al. combined WGCNA with machine learning to identify aging-related key genes associated with COPD, constructed a neural network-based diagnostic model, and validated their findings using single-cell data (25). In addition, Liao et al. integrated bulk RNA sequencing and single-cell RNA sequencing data to explore the roles of RNA methylation and autophagy pathways in COPD (26). Peng et al. used WGCNA and machine learning algorithms to identify mitochondrial function-related COPD biomarkers and analyzed their correlation with immune infiltration (27). Although these studies have enriched our understanding of COPD pathogenesis to some extent, most focus on the whole transcriptome or specific pathways and lack systematic investigations targeting inflammation-related genes, as well as integration of single-cell data and clinical subtypes. This study focuses on inflammation-related genes in COPD. Transcriptome data were used to identify differentially expressed inflammation-related genes between COPD patients and healthy controls, and their potential roles in disease were explored through functional and pathway enrichment analyses. Furthermore, key feature genes were screened using multiple machine learning algorithms to construct a multigene risk prediction model. Immune infiltration analysis and single-cell transcriptomic data were integrated to characterize the involvement of these genes in disease progression and inflammatory subtype differentiation. Additionally, pathway activity differences among inflammatory subtypes were investigated, and potential traditional Chinese medicine (TCM) monomers targeting inflammation-related genes were screened. Through this multi-dimensional and multi-layered approach, the study aims to deepen the understanding of inflammatory mechanisms in COPD, broaden strategies for individualized intervention, and provide theoretical support for precision clinical management.

## 2 Methods

### 2.1 Acquisition and differential gene analysis of COPD transcriptome data

This study downloaded transcriptome data of airway epithelial tissues from COPD patients and normal individuals from the Gene Expression Omnibus (GEO) database and performed differential gene expression analysis using R software and its limma package to reveal differences in gene expression between COPD patients and healthy individuals. The transcriptome data included control and experimental groups, each with no fewer than three samples, and restricted to data from humans. Gene expression data were normalized and differentially analyzed using the limma package. Significantly differentially expressed genes were selected based on an absolute logFC > 0.585 and an adjusted *p*-value < 0.05. The transcriptome dataset used in this analysis was GSE21359, which comprises gene expression profiles from small airway epithelial tissues collected via fiberoptic bronchoscopy. A total of 135 samples were included: 53 from healthy nonsmokers, 59 from healthy smokers, and 23 from smokers with COPD. All samples were processed on the Affymetrix Human Genome U133 Plus 2.0 Array platform (GPL570).

### 2.2 Functional and pathway enrichment analysis of differential genes

To reveal the functional and pathway enrichment of differential genes between COPD patients and normal individuals, this study conducted Gene Ontology (GO), Kyoto Encyclopedia of Genes and Genomes (KEGG), and Gene Set Enrichment Analysis (GSEA). GO analysis categorizes genes into biological processes, cellular components, and molecular functions to provide insights into the roles these genes may play in cellular processes. KEGG enrichment analysis identifies pathways that these genes are involved in, revealing their potential impact on disease mechanisms. GSEA allows for the identification of enriched biological pathways or gene sets based on gene expression data, providing a higher-level understanding of biological functions.

All data processing and analyses were completed using R software. After converting differential gene symbols to gene IDs, GO enrichment analyses were performed using the enrichGO functions in the clusterProfiler package. The KEGG enrichment analysis results were obtained from the DAVID database. For further exploration of functional enrichment, GSEA was performed using the GSEA function in the clusterProfiler package and the gene set file h.all.v2022.1.Hs.symbols.gmt. The screening criteria for enrichment analysis results were p.adjust < 0.05.

### 2.3 Screening and pathway analysis of inflammation-related differential genes

Inflammation is a key factor in COPD pathogenesis, and genes involved in inflammatory responses were selected from existing literature. These inflammatory genes were then intersected with the differentially expressed genes to identify inflammation-related genes specific to COPD. Correlation analysis was performed to investigate the relationships between these genes, aiming to uncover potential interactions and identify genes that may work synergistically in COPD inflammation. Pearson correlation coefficients were calculated to quantify the linear relationship between gene expression profiles using the cor() function in R software. The igraph package in R software was used to visualize these interactions as a network, where the edges represent gene–gene correlations, with blue edges indicating positive correlations and red edges representing negative correlations. Edge width was proportional to the strength of the correlation. Pathway enrichment analysis of inflammation-related genes was conducted using the Metascape database, which aggregates functional annotation and enrichment results from multiple sources.

### 2.4 Machine learning screening of COPD inflammatory feature genes

To identify key inflammation-related genes that could serve as features for COPD, machine learning approaches were employed. Lasso regression was used to identify a subset of important genes by shrinking less important variables to zero. This was achieved using the glmnet package in R software. Cross-validation was applied to determine the optimal regularization parameter, ensuring that the model does not overfit the data. Additionally, a random forest model was used to calculate feature importance scores for each gene using the randomForest package in R. Genes with importance scores greater than 2 were selected as significant features. A Venn diagram was used to visually represent the overlap of feature genes identified by both methods.

### 2.5 Construction and validation of COPD risk prediction model

To further validate the clinical application value of inflammation-related COPD feature genes, a nomogram model was constructed using logistic regression analysis. The rms and rmda packages in R software were employed for model development. Nomograms are graphical representations of statistical models that calculate the probability of a clinical event. Gene expression data were first processed, and the expression levels of the selected feature genes were extracted. A logistic regression model was built using the lrm() function from the rms package, with disease risk as the dependent variable and the selected feature genes as independent variables. The nomogram was then generated using the nomogram() function, with predicted probabilities plotted against disease risk. To evaluate the accuracy of the model, calibration curves were constructed using the calibrate() function, which applies bootstrap method (B = 1,000) to assess prediction reliability. Decision curve analysis was performed to assess the clinical benefit of the model at various threshold probabilities. The decision_curve() function from the rmda package was used to analyze the clinical net benefit of the model by plotting threshold probabilities against expected benefits, allowing for the evaluation of model performance under different clinical scenarios.

### 2.6 Expression analysis of inflammation-related feature genes across GOLD stages in COPD

To systematically investigate the expression patterns of inflammation-related feature genes at different stages of COPD, this study retrieved gene expression datasets containing GOLD staging information from the GEO database, including normal control samples and COPD patients at GOLD stages 1 through 4. The dataset used for this analysis was GSE47460. The raw expression matrix was first normalized. Subsequently, the standardized gene expression data and corresponding GOLD stage information were extracted. The Wilcoxon rank-sum test was used to statistically evaluate expression differences between each GOLD stage (GOLD 1, 2, 3, and 4) and the normal control group. Additionally, pairwise comparisons between GOLD stages were performed to identify expression trends associated with disease progression. Data visualization was conducted using the ggplot2 and ggpubr packages in R, with box plots combined with dot plots to illustrate the distribution and statistical significance of gene expression across different COPD stages.

### 2.7 Immune microenvironment and single-cell sequencing analysis

The immune microenvironment of COPD patients was analyzed using the ESTIMATE algorithm and the CIBERSORT method. ESTIMATE calculates the immune and stromal scores of a sample, which provide insight into the relative abundance of immune and non-immune cells in the tissue. The CIBERSORT method estimates the proportion of 22 immune cell types in each sample, providing a detailed analysis of immune infiltration in COPD. Single-cell RNA sequencing data from COPD patients’ lung tissues were processed using the Seurat package in R to explore the gene expression profiles at the single-cell level. The single-cell RNA sequencing data analyzed in this study were obtained from the GEO dataset GSE167295, which includes 29,961 cells isolated from peripheral lung parenchymal tissues. The dataset comprises samples from three patients with severe COPD. All samples were derived from human whole lung tissues and were sequenced using the Illumina NextSeq 500 platform (28). Principal component analysis was used for dimensionality reduction, and t-SNE clustering was applied to visualize the distinct cell populations in the lung tissue. A dot plot was used to visualize the expression of inflammation-related genes in different cell types, providing insights into the specific roles of these genes in COPD pathogenesis.

### 2.8 Clustering and analysis of COPD inflammatory subtypes

To better understand the heterogeneity of inflammation in COPD, consensus clustering was performed to identify distinct inflammatory subtypes within the COPD patient population. This method utilizes the ConsensusClusterPlus R package to perform consensus clustering on inflammation-related COPD feature genes expression data. The data is first filtered to retain only COPD group samples. K-means clustering with Euclidean distance is applied, and the clustering process is repeated 50 times to ensure stability. The maximum number of clusters is set to 9. The calcICL function is used to compute consensus scores. After determining the optimal number of clusters, the clustering results are extracted and combined with the gene expression data for final output. Subsequently, five independent GEO datasets (GSE11906, GSE37768, GSE151052, GSE162635, and GSE8581) and GSE21359 were used for external validation of the upregulated inflammation-related COPD feature genes in the inflammatory subtypes by calculating the standardized mean difference.

This study employed Gene Set Variation Analysis (GSVA) to investigate the differences in pathway activation between various inflammatory subtypes of COPD patients. The analysis was based on three gene sets: c2.cp.kegg.symbols.gmt, c5.go.symbols.gmt, and hall.v2022.1.Hs.symbols.gmt. The R packages reshape2, ggpubr, limma, GSEABase, and GSVA were used for data preprocessing, GSVA analysis, differential analysis, and visualization. The expression data were first standardized, and only the COPD group samples were retained for further analysis. GSVA was performed using the gsva() function, which calculated the GSVA scores for each sample. These scores were then normalized. Based on the clustering results, the GSVA scores for COPD samples in different inflammatory subtypes were extracted. A *t*-test was performed to identify significantly different pathways between the subtypes, and pathways were classified as upregulated or downregulated based on *p*-values and t-statistics. Finally, the top 10 and bottom 10 most significantly different pathways were selected and visualized using bar plots, offering further insights into the pathway activation characteristics across the different inflammatory subtypes of COPD. To explore the differences in the immune microenvironment between COPD patients with different inflammatory subtypes, this study integrated immune cell infiltration data from COPD samples with clustering results. Statistical comparisons of immune cell infiltration across the different inflammatory subtypes were conducted using *t*-tests. Box plots were then employed to illustrate the distribution of immune cell infiltration among the various subtypes.

### 2.9 Screening of traditional Chinese medicine monomer compounds, molecular docking, and molecular dynamics simulation analysis

To identify TCM monomer compounds with potential therapeutic value, this study utilized the HERB database to screen for candidates targeting inflammation-related genes associated with COPD. Molecular docking simulations were subsequently performed to predict the binding affinity between the selected TCM compounds and key COPD-related biomarker proteins. The structural data for TCM compounds and target proteins were obtained from the PubChem database and the RCSB Protein Data Bank, respectively. Molecular docking was conducted using AutoDock and PyMOL software, which are widely used tools for simulating interactions between small molecules and proteins. The results of the docking simulations facilitated the identification of candidate compounds with favorable binding affinity, providing a potential pharmacological basis for targeting inflammation-related genes in COPD therapy.

To further verify the binding stability and interaction mechanisms between active TCM compounds and key COPD target proteins, molecular dynamics simulations were performed on the constructed compound–protein complexes using GROMACS 2023.2. The simulation systems were parameterized using the CHARMM36 force field with the TIP3P water model, and Na^+^ and Cl^−^ ions were added to neutralize the system charge. Energy minimization was performed in two stages using the steepest descent and conjugate gradient methods. This was followed by 100 ps of NVT and NPT equilibration, employing the V-rescale thermostat (at 300 K) and the Parrinello–Rahman barostat (at 1 bar), respectively. Based on the equilibrated structures, a 100 ns production simulation was carried out with a time step of 2 fs. Long-range electrostatic interactions were treated using the Particle Mesh Ewald method, and both van der Waals and Coulomb interactions were truncated at 1.0 nm. After the simulation, structural parameters including root-mean-square deviation (RMSD), root-mean-square fluctuation (RMSF), radius of gyration (Rg), solvent-accessible surface area (SASA), and the number of hydrogen bonds were calculated to assess the stability of the complexes.

## 3 Results

### 3.1 Significantly differentially expressed genes between COPD patients and normal individuals

**Figure 1:**
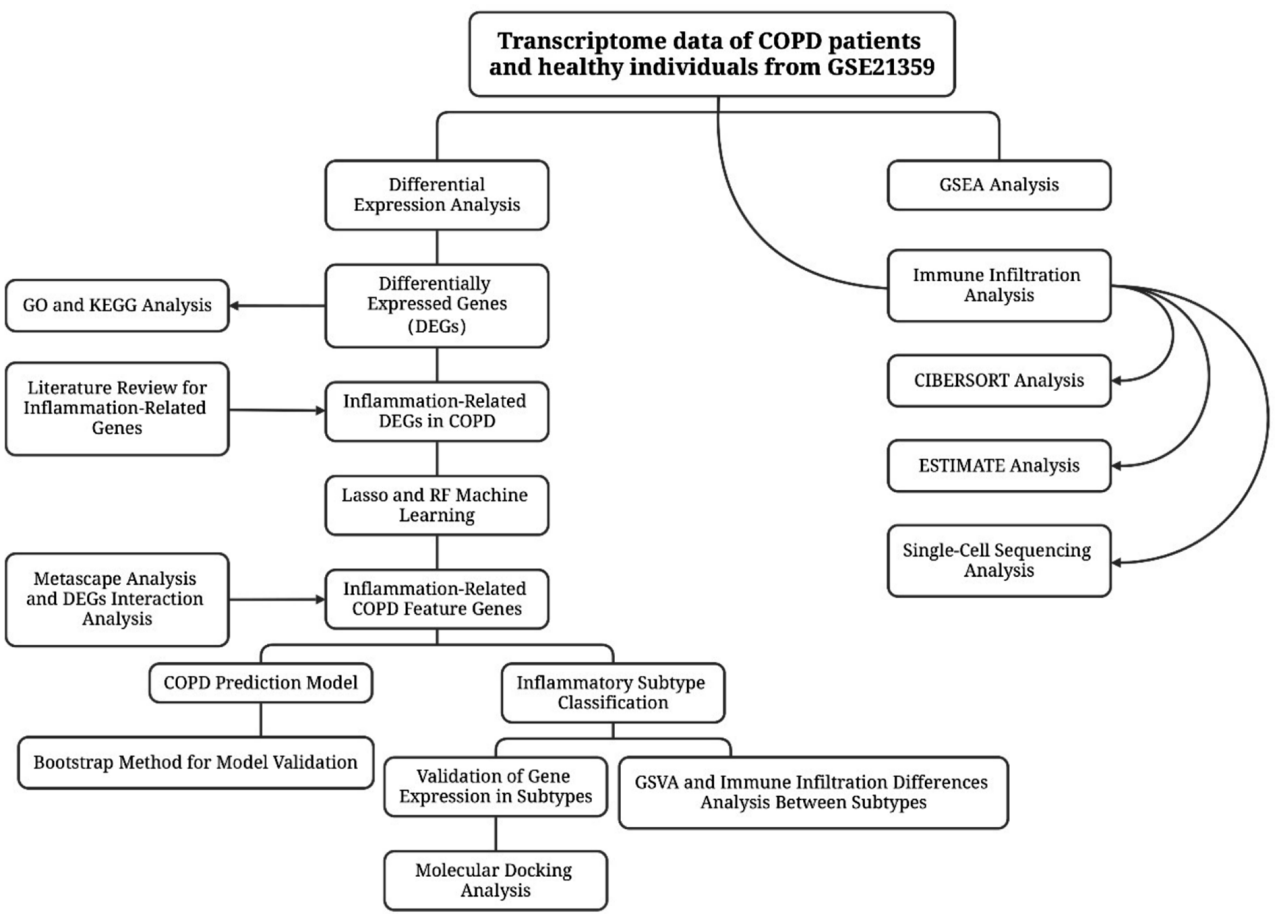
The workflow of the entire study.

**Figure 2:**
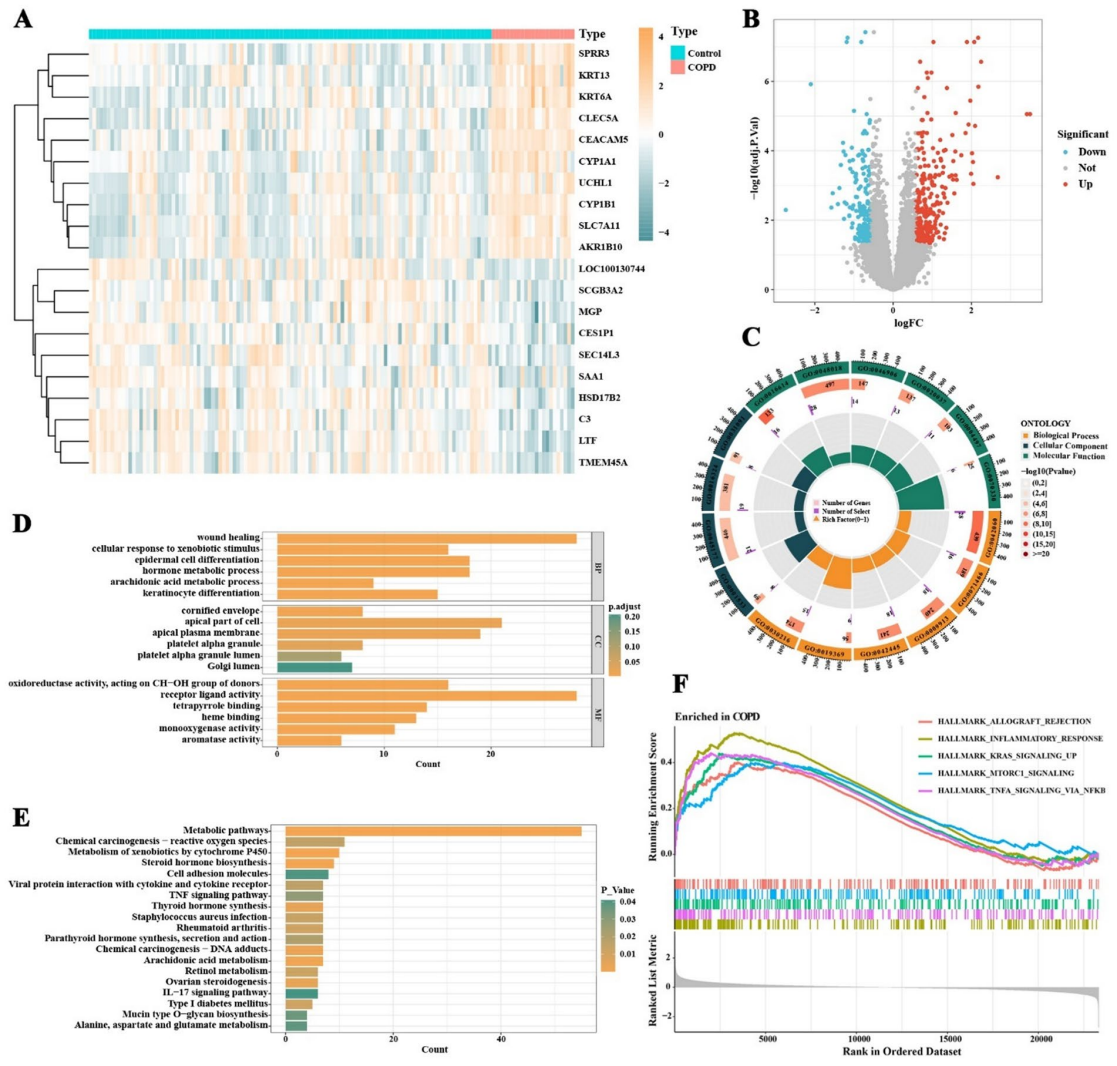
Differential gene expression and enrichment pathway analysis in COPD patients and normal individuals. **(A)** Expression patterns of differentially expressed genes in COPD patients and normal individuals. **(B)** Significance and fold changes of gene expression. **(C)** GO enrichment analysis results covering biological processes, cellular components, and molecular functions. **(D)** GO enrichment analysis shows significant enrichment of differentially expressed genes in processes such as keratinocyte differentiation, arachidonic acid metabolism, hormone metabolism, epidermal cell differentiation, response to xenobiotic stimulus, and wound healing. **(E)** KEGG pathway enrichment analysis reveals significant enrichment of genes in pathways such as arachidonic acid metabolism, IL-17 signaling pathway, TNF signaling pathway, cell adhesion molecules, retinol metabolism, and cytochrome P450 metabolism. **(F)** GSEA analysis suggests significant enrichment of pathways such as allograft rejection, inflammatory response, and TNF-α signaling via NF-κB in the small airway epithelial tissues of COPD patients.

The research workflow is shown in Figure 1. After searching the GEO database with specific criteria, the GSE21359 dataset was selected for further analysis. This dataset includes a total of 135 samples from airway epithelial cells: 53 healthy nonsmokers, 59 healthy smokers, and 23 smokers with clinically diagnosed COPD. Subject metadata revealed a wide range of smoking exposures (e.g., 0.5 to 119 pack-years), and the COPD group included GOLD stages I to III. The average age across groups ranged from 21 to 73 years, with both male and female participants represented. Detailed demographic and clinical parameters are provided in Table 1. Standardization and differential analysis of the above expression data using the limma package identified a total of 495 significantly differentially expressed genes (Supplementary Table 1). These genes may play crucial roles in the molecular mechanisms underlying COPD and could potentially serve as biomarkers for the disease. Figure 2A shows the expression patterns of some differentially expressed genes. Figure 2B displays the significance and fold changes of gene expression. In the volcano plot, red dots represent significantly upregulated genes, while blue dots represent significantly downregulated genes. This analysis clearly highlights the contrast in gene expression between COPD patients and healthy controls, providing insights into the pathogenesis of COPD.

**Table 1:** Demographic information, smoking history, and GOLD classification of subjects included in the GSE21359 airway epithelial transcriptome dataset.

Sample ID	Source	Sex	Smoking status	Age
GSM101095	Airway epithelial cells	Male	Non-smoker	41
GSM101096	Airway epithelial cells	Male	Non-smoker	35
GSM101097	Airway epithelial cells	Male	Non-smoker	61
GSM101098	Airway epithelial cells	Female	Non-smoker	37
GSM101100	Airway epithelial cells	Male	Non-smoker	47
GSM101101	Airway epithelial cells	Male	Non-smoker	38
GSM101102	Airway epithelial cells	Female	Non-smoker	49
GSM101103	Airway epithelial cells	Male	Non-smoker	45
GSM101104	Airway epithelial cells	Male	Non-smoker	36
GSM101105	Airway epithelial cells	Male	Non-smoker	38
GSM101106	Airway epithelial cells	Male	Non-smoker	35
GSM101107	Airway epithelial cells	Male	Smoker, 21 pack-years	46
GSM101111	Airway epithelial cells	Female	Smoker, 23 pack-years	37
GSM101113	Airway epithelial cells	Male	Smoker, 28 pack-years	45
GSM101114	Airway epithelial cells	Male	Smoker, 20 pack-years	48
GSM101115	Airway epithelial cells	Male	Smoker, 38 pack-years	50
GSM101116	Airway epithelial cells	Female	Smoker, 23 pack-years	46
GSM114089	Airway epithelial cells	Male	Smoker, 80 pack-years	56
GSM114090	Airway epithelial cells	Male	Smoker, 60 pack-years	59
GSM190149	Airway epithelial cells	Male	Non-smoker	49
GSM190150	Airway epithelial cells	Male	Non-smoker	34
GSM190151	Airway epithelial cells	Male	Non-smoker	44
GSM190152	Airway epithelial cells	Male	Non-smoker	45
GSM190153	Airway epithelial cells	Female	Non-smoker	45
GSM190154	Airway epithelial cells	Female	Non-smoker	29
GSM190155	Airway epithelial cells	Male	Non-smoker	42
GSM190156	Airway epithelial cells	Male	Non-smoker	56
GSM252828	Airway epithelial cells	Male	COPD, GOLD-I, 50 pack-years	47
GSM252829	Airway epithelial cells	Male	COPD, GOLD-II, 33 pack-years	47
GSM252830	Airway epithelial cells	Male	COPD, GOLD-II, 35 pack-years	50
GSM252831	Airway epithelial cells	Male	COPD, GOLD-II, 20 pack-years	55
GSM252833	Airway epithelial cells	Male	COPD, GOLD-I, 48 pack-years	59
GSM252835	Airway epithelial cells	Male	COPD, GOLD-II, 75 pack-years	51
GSM252836	Airway epithelial cells	Male	COPD, GOLD-II, 27 pack-years	46
GSM252837	Airway epithelial cells	Male	COPD, GOLD-II, 60 pack-years	56
GSM252838	Airway epithelial cells	Male	COPD, GOLD-III, 110 pack-years	60
GSM252839	Airway epithelial cells	Male	COPD, GOLD-I, 22 pack-years	46
GSM252841	Airway epithelial cells	Male	COPD, GOLD-I, 23 pack-years	52
GSM252871	Airway epithelial cells	Male	Smoker, 24 pack-years	40
GSM252876	Airway epithelial cells	Male	Smoker, 24 pack-years	45
GSM252879	Airway epithelial cells	Male	Smoker, 20 pack-years	41
GSM252880	Airway epithelial cells	Male	Smoker, 29 pack-years	47
GSM252881	Airway epithelial cells	Male	Smoker, 45 pack-years	41
GSM252882	Airway epithelial cells	Male	Smoker, 32 pack-years	48
GSM252884	Airway epithelial cells	Female	Smoker, 36 pack-years	43
GSM252885	Airway epithelial cells	Male	Smoker, 15 pack-years	41
GSM254149	Airway epithelial cells	Female	Non-smoker	41
GSM254150	Airway epithelial cells	Male	Non-smoker	35
GSM254151	Airway epithelial cells	Male	Non-smoker	37
GSM254152	Airway epithelial cells	Male	Non-smoker	31
GSM254157	Airway epithelial cells	Male	Smoker, 23 pack-years	45
GSM254158	Airway epithelial cells	Female	Smoker, 22 pack-years	50
GSM254159	Airway epithelial cells	Female	Smoker, 33 pack-years	46
GSM254160	Airway epithelial cells	Male	Smoker, 16 pack-years	49
GSM254161	Airway epithelial cells	Female	Smoker, 47 pack-years	40
GSM254163	Airway epithelial cells	Female	COPD, GOLD-II, 27.5 pack-years	51
GSM254169	Airway epithelial cells	Female	COPD, GOLD-II, 34 pack-years	48
GSM254172	Airway epithelial cells	Female	COPD, GOLD-II, 15 pack-years	53
GSM254173	Airway epithelial cells	Male	COPD, GOLD-II, 29 pack-years	42
GSM254174	Airway epithelial cells	Male	COPD, GOLD-I, 32.5 pack-years	36
GSM254175	Airway epithelial cells	Male	COPD, GOLD-I, 14 pack-years	44
GSM254176	Airway epithelial cells	Male	COPD, GOLD-I, 24 pack-years	62
GSM298219	Airway epithelial cells	Male	Non-smoker	44
GSM298220	Airway epithelial cells	Male	Non-smoker	60
GSM298221	Airway epithelial cells	Male	Non-smoker	49
GSM298222	Airway epithelial cells	Female	Non-smoker	36
GSM298223	Airway epithelial cells	Male	Non-smoker	38
GSM298224	Airway epithelial cells	Male	Non-smoker	73
GSM298225	Airway epithelial cells	Male	Non-smoker	49
GSM298226	Airway epithelial cells	Female	Non-smoker	22
GSM298227	Airway epithelial cells	Male	Non-smoker	29
GSM298228	Airway epithelial cells	Female	Non-smoker	39
GSM298229	Airway epithelial cells	Female	Non-smoker	48
GSM298230	Airway epithelial cells	Male	Smoker, 30 pack-years	39
GSM298231	Airway epithelial cells	Female	Smoker, 45 pack-years	54
GSM298232	Airway epithelial cells	Male	Smoker, 30 pack-years	43
GSM298233	Airway epithelial cells	Male	Smoker, 3 pack-years	36
GSM298234	Airway epithelial cells	Female	Smoker, 22.5 pack-years	41
GSM298235	Airway epithelial cells	Female	Smoker, 19 pack-years	46
GSM298236	Airway epithelial cells	Male	Smoker, 11 pack-years	47
GSM298237	Airway epithelial cells	Male	Smoker, 12 pack-years	41
GSM298238	Airway epithelial cells	Female	Smoker, 20 pack-years	42
GSM298239	Airway epithelial cells	Male	Smoker, 26 pack-years	46
GSM298240	Airway epithelial cells	Male	Smoker, 13 pack-years	41
GSM298241	Airway epithelial cells	Female	Smoker, 7.6 pack-years	32
GSM298242	Airway epithelial cells	Female	Smoker, 3.8 pack-years	27
GSM298243	Airway epithelial cells	Male	Smoker, 5 pack-years	35
GSM298244	Airway epithelial cells	Male	Smoker, 44.3 pack-years	40
GSM298245	Airway epithelial cells	Male	Smoker, 43 pack-years	48
GSM298246	Airway epithelial cells	Male	Smoker, 33 pack-years	47
GSM298247	Airway epithelial cells	Male	Smoker, 38 pack-years	41
GSM300859	Airway epithelial cells	Female	Non-smoker	62
GSM302396	Airway epithelial cells	Male	Non-smoker	47
GSM302397	Airway epithelial cells	Male	Non-smoker	39
GSM302399	Airway epithelial cells	Female	Smoker, 38 pack-years	27
GSM350871	Airway epithelial cells	Male	Non-smoker	24
GSM350873	Airway epithelial cells	Male	Non-smoker	31
GSM350874	Airway epithelial cells	Female	Smoker, 17.5 pack-years	43
GSM350955	Airway epithelial cells	Male	Non-smoker	26
GSM350956	Airway epithelial cells	Female	Non-smoker	33
GSM350957	Airway epithelial cells	Male	Smoker, 46 pack-years	45
GSM350958	Airway epithelial cells	Female	Smoker, 26.5 pack-years	48
GSM364037	Airway epithelial cells	Female	COPD, GOLD-II, 38.5 pack-years	57
GSM364038	Airway epithelial cells	Male	COPD, GOLD-I, 119 pack-years	66
GSM364041	Airway epithelial cells	Male	COPD, GOLD-I, 26 pack-years	45
GSM364045	Airway epithelial cells	Male	COPD, GOLD-II, 24 pack-years	45
GSM364046	Airway epithelial cells	Female	Smoker, 0.5 pack-years	48
GSM364048	Airway epithelial cells	Female	Smoker, 56.5 pack-years	47
GSM410161	Airway epithelial cells	Female	Non-smoker	21
GSM410162	Airway epithelial cells	Male	Non-smoker	45
GSM410163	Airway epithelial cells	Male	Non-smoker	55
GSM410164	Airway epithelial cells	Male	Smoker, 45 pack-years	47
GSM410165	Airway epithelial cells	Male	Smoker, 11 pack-years	39
GSM434049	Airway epithelial cells	Male	Non-smoker	68
GSM434050	Airway epithelial cells	Female	Non-smoker	26
GSM434051	Airway epithelial cells	Female	Non-smoker	45
GSM434052	Airway epithelial cells	Male	Non-smoker	40
GSM434053	Airway epithelial cells	Male	Smoker, 29 pack-years	40
GSM434054	Airway epithelial cells	Male	Smoker, 47 pack-years	46
GSM434055	Airway epithelial cells	Male	Smoker, 19.5 pack-years	47
GSM434056	Airway epithelial cells	Male	Smoker, 27 pack-years	29
GSM434057	Airway epithelial cells	Male	Smoker, 10 pack-years	30
GSM434058	Airway epithelial cells	Male	Smoker, 24 pack-years	47
GSM434059	Airway epithelial cells	Female	Smoker, 71 pack-years	43
GSM434060	Airway epithelial cells	Male	Smoker, 46 pack-years	48
GSM434061	Airway epithelial cells	Female	Smoker, 10.5 pack-years	24
GSM434062	Airway epithelial cells	Female	Smoker, 1 pack-years	27
GSM434063	Airway epithelial cells	Male	Smoker, 26 pack-years	54
GSM434064	Airway epithelial cells	Female	COPD, GOLD-III, 53 pack-years	73
GSM458579	Airway epithelial cells	Male	Non-smoker	27
GSM458580	Airway epithelial cells	Male	Non-smoker	34
GSM458581	Airway epithelial cells	Male	Non-smoker	27
GSM458582	Airway epithelial cells	Female	Non-smoker	47
GSM469991	Airway epithelial cells	Male	Non-smoker	37
GSM470000	Airway epithelial cells	Male	Smoker, 51 pack-years	48

### 3.2 Significantly enriched biological processes and pathways of differential genes in COPD

Through GO, KEGG, and GSEA enrichment analyses of the differentially expressed genes in COPD, this study identified multiple biological processes and signaling pathways closely associated with COPD pathogenesis. GO enrichment analysis revealed that the differentially expressed genes were significantly enriched in processes such as keratinocyte differentiation, arachidonic acid metabolism, hormone metabolism, epidermal cell differentiation, response to xenobiotic stimulus, fatty acid metabolism, and wound healing (Figures 2C,D; Supplementary Table 2), suggesting widespread abnormalities in epithelial structural maintenance, inflammatory mediator synthesis, and metabolic homeostasis in COPD patients. KEGG pathway analysis further indicated significant enrichment of COPD-related genes in pathways including arachidonic acid metabolism, IL-17 signaling, TNF signaling, cell adhesion molecules, retinol metabolism, cytochrome P450 metabolism, and mucin-type O-glycan biosynthesis (Figure 2E; Supplementary Table 3). These findings reflect the multi-layered regulation of airway inflammation, immune modulation, epithelial barrier function, and mucus secretion in COPD. GSEA results further demonstrated significant activation of inflammatory response, TNF-*α* signaling via NF-κB, and allograft rejection pathways in the COPD group, along with notable upregulation of KRAS signaling, mTORC1 signaling, epithelial–mesenchymal transition (EMT), and early and late estrogen response pathways (Figure 2F; Supplementary Table 4). Overall, the pathogenesis of COPD involves not only immune inflammation but also dysregulation of metabolic processes, structural remodeling, and endocrine signaling. Among these, inflammation serves as the central pathological mechanism driving the persistent progression of COPD.

### 3.3 Pathway enrichment of inflammation-related COPD genes

**Figure 3:**
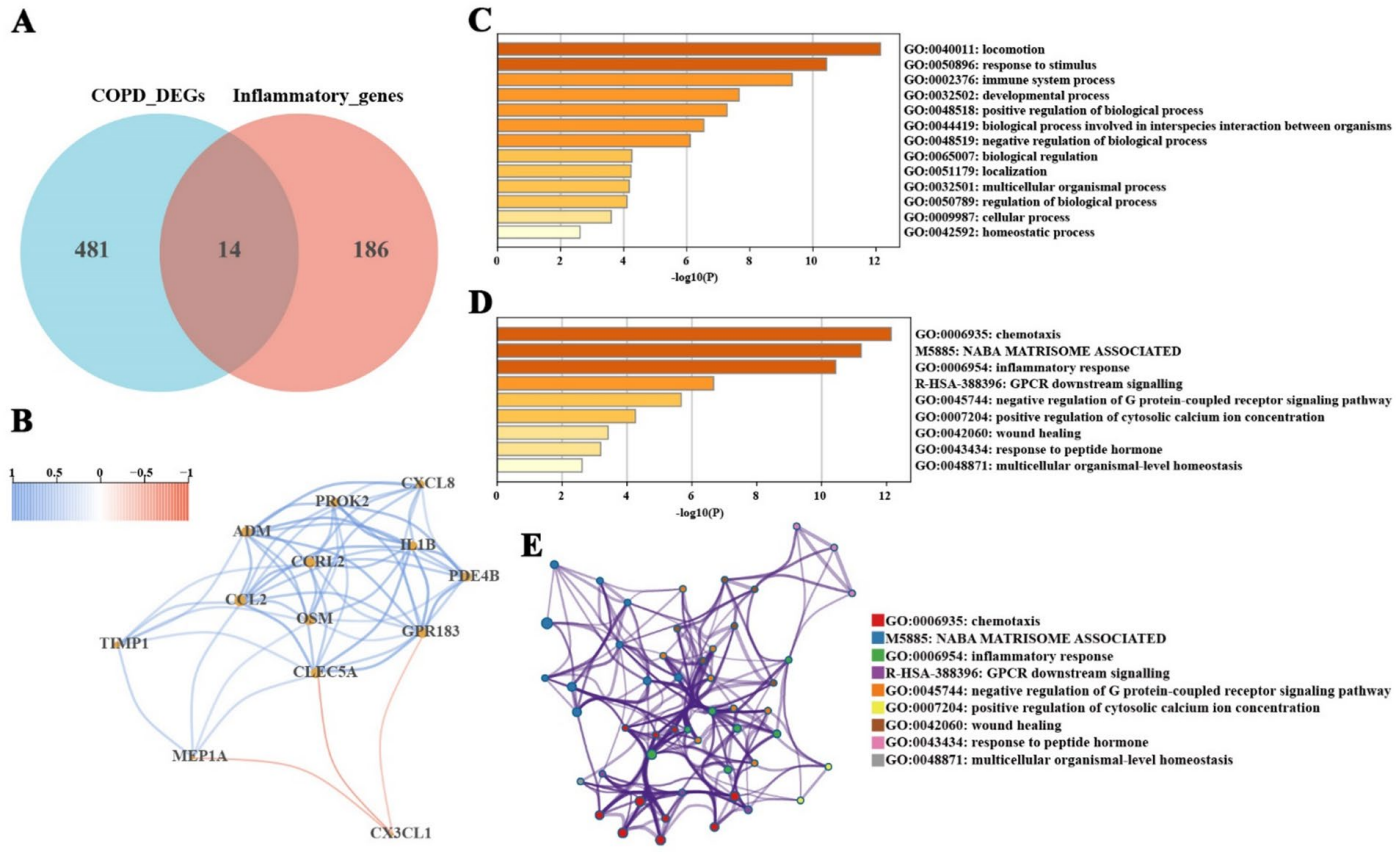
Screening and pathway enrichment analysis of inflammation-related COPD genes. **(A)** Intersection of significantly differentially expressed genes in COPD and known inflammatory genes, identifying 14 inflammation-related genes. **(B)** Interaction relationships among inflammation-related genes. **(C,D)** Functional enrichment analysis of inflammation-related genes using the Metascape database, showing significant enrichment in immune system processes, response to stimulus, inflammatory response, chemotaxis, and wound healing pathways. **(E)** Functional cluster analysis using the Metascape database reveals potential biological links between functional clusters.

To investigate the role of inflammation in COPD, this study intersected inflammation-related genes with the significantly differentially expressed genes in COPD, resulting in the identification of 14 common genes (Figure 3A; Supplementary Table 5). These genes represent key regulators of the inflammatory microenvironment in COPD and are potentially therapeutic targets. Correlation analysis revealed that most of these inflammation-related genes interact with each other (Figure 3B), suggesting that they may function synergistically in the inflammatory response of COPD. Pathway enrichment analysis of these genes was performed using the Metascape database, and the results demonstrated significant enrichment in immune system processes and responses to stimuli, influencing pathways related to inflammation, chemotaxis, wound healing, and G-protein-coupled receptor signaling (Figures 3C,D). The relationships between functional clusters are illustrated in Figure 3E, highlighting potential biological connections among these clusters. These findings further emphasize the critical role of inflammation in the pathogenesis and progression of COPD.

### 3.4 Screening and diagnostic efficiency of COPD inflammatory feature genes

**Figure 4:**
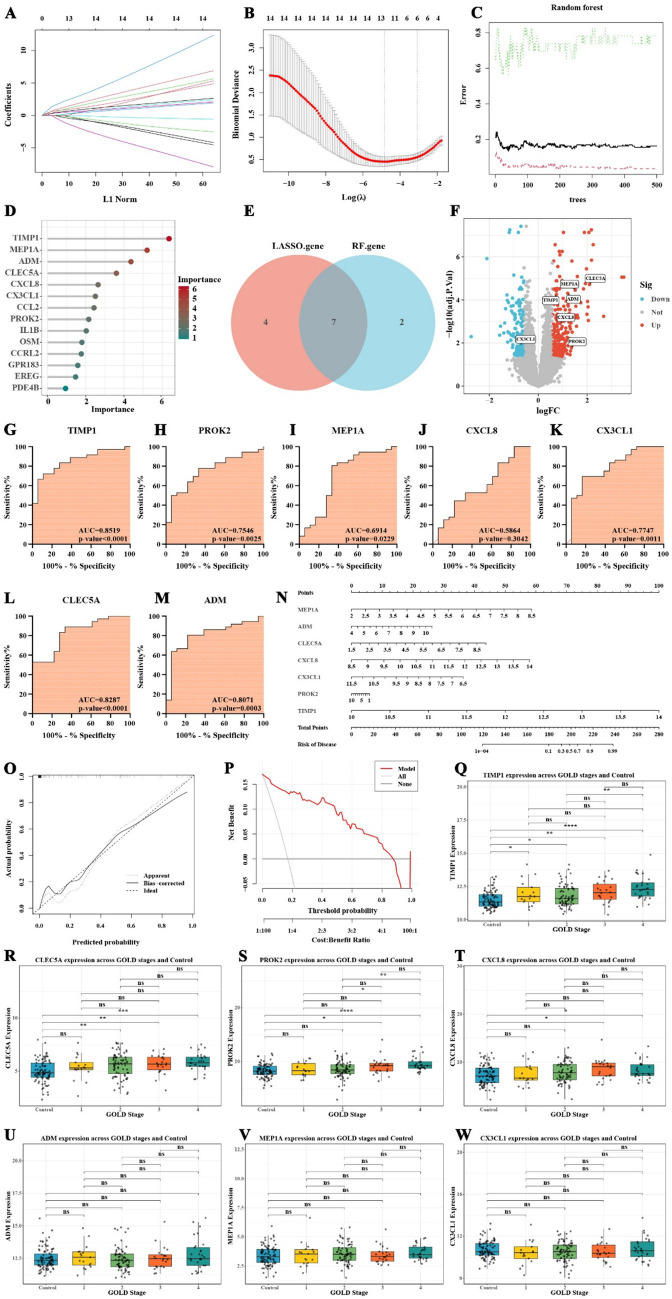
Screening, diagnostic efficiency, and construction of risk prediction model for COPD inflammatory feature genes. **(A)** Lasso regression model showing gene coefficient changes. **(B)** Cross-validation results of Lasso regression. **(C)** Prediction error analysis from the random forest model. **(D)** Gene importance scores from the random forest algorithm. **(E)** Venn diagram showing the overlap of genes identified by Lasso and random forest. **(F)** Volcano plot of the expression changes of inflammation-related feature genes in COPD. **(G–M)** ROC curve analysis of inflammation-related feature genes in COPD. **(N)** Nomogram for predicting COPD risk based on inflammatory feature genes. **(O)** Calibration curve of the COPD risk prediction model. **(P)** Decision curve analysis assessing the model’s clinical benefit. **(Q–W)** Boxplots comparing the expression of inflammation-related feature genes between the normal group and GOLD stages 1–4, as well as among the different GOLD stages (* *p* < 0.05, ** *p* < 0.01, *** *p* < 0.001).

This study employed Lasso regression and random forest algorithms to identify inflammation-related feature genes in COPD. Lasso regression analysis was first conducted to determine the optimal regularization parameter and to screen for significant feature genes. The variations in gene coefficients across different regularization parameters in the Lasso model are illustrated in Figure 4A, while the cross-validation results are presented in Figure 4B. The feature genes identified through Lasso regression included CLEC5A, MEP1A, ADM, TIMP1, CXCL8, EREG, CX3CL1, PROK2, OSM, GPR183, and CCRL2. Subsequently, the random forest algorithm was applied to further refine the feature gene selection by calculating their importance scores. The prediction errors for various decision tree numbers in the random forest model are shown in Figure 4C, and the importance scores for each gene are displayed in Figure 4D. The random forest analysis identified TIMP1, MEP1A, ADM, CLEC5A, CXCL8, CX3CL1, CCL2, PROK2, and IL1B as key feature genes.

Based on the results from both methods, we found that TIMP1, MEP1A, ADM, CLEC5A, CXCL8, CX3CL1, and PROK2 exhibited high importance (Figure 4E). Notably, CX3CL1 was downregulated in COPD patients, while the remaining six genes were upregulated (Figure 4F). The ROC curve analysis revealed that TIMP1 (AUC = 0.8519, *p*-value < 0.0001), CLEC5A (AUC = 0.8287, *p*-value < 0.0001), and ADM (AUC = 0.8071, *p*-value = 0.0003) had AUC values exceeding 0.8 and highly significant *p*-values, indicating their high diagnostic efficiency in distinguishing COPD patients from non-COPD individuals (Figures 4G–M).

### 3.5 Construction of COPD risk prediction model based on inflammatory feature genes

To further evaluate the clinical utility of inflammation-related feature genes in COPD, a nomogram model was developed to predict the risk of disease occurrence. This nomogram incorporated seven inflammation-related COPD feature genes: MEP1A, ADM, CLEC5A, CXCL8, CX3CL1, PROK2, and TIMP1. The score for each gene in the nomogram was used to calculate the total score, which corresponds to the predicted risk of COPD (Figure 4N). The results highlight the significant role of these seven genes in predicting the risk of COPD development.

Calibration curve analysis revealed that, after bias correction, the predicted risk of COPD occurrence was relatively accurate (Figure 4O). Additionally, decision curve analysis demonstrated that, within the threshold range of 0.1 to 0.7, the use of the nomogram model to predict COPD risk provides meaningful clinical benefits (Figure 4P). These findings underscore the clinical applicability of the selected inflammation-related feature genes in predicting the risk of COPD.

### 3.6 Expression changes of inflammation-related feature genes during COPD progression

To further investigate the expression patterns of seven inflammation-related feature genes across different stages of COPD and their potential value in disease classification, we analyzed the GSE47460 dataset, which includes 91 normal controls and 140 COPD patients classified into GOLD stages 1–4. Supplementary Table 6 provides an overview of the clinical characteristics of the subjects included in this dataset. The Wilcoxon rank-sum test was employed to assess differences between each COPD stage and the normal group, as well as among the GOLD stages. The results revealed that TIMP1 expression was significantly elevated in all COPD stages compared to the control group (GOLD_1: *p* = 0.0416; GOLD_2: *p* = 0.0179; GOLD_3: *p* = 0.00171; GOLD_4: *p* = 8.02 × 10^−7^; Figure 4Q). Moreover, TIMP1 expression in GOLD_4 was markedly higher than in GOLD_2 (*p* = 0.00139; Figure 4Q), indicating a progressive upregulation with disease advancement. CLEC5A expression was significantly increased in GOLD_2 (*p* = 0.00148), GOLD_3 (*p* = 0.00344), and GOLD_4 (*p* = 0.000262) relative to controls, although no significant differences were observed among the GOLD stages themselves (Figure 4R). This suggests a general upward trend without stage-specific variation. PROK2 expression was significantly elevated in GOLD_3 (*p* = 0.0147) and GOLD_4 (*p* = 2.15 × 10^−5^) compared to the control group (Figure 4S). Furthermore, its expression in GOLD_4 was markedly higher than in GOLD_1 (*p* = 0.0257) and GOLD_2 (*p* = 0.00285), indicating a sustained upregulation of PROK2 during advanced stages of COPD (Figure 4S). Similarly, CXCL8 expression was significantly higher in GOLD_3 (*p* = 0.0235) and GOLD_4 (*p* = 0.0284) than in controls, suggesting its increased activity in moderate to severe COPD (Figure 4T). In contrast, ADM, MEP1A, and CX3CL1 did not exhibit significant expression changes across COPD stages, implying that these genes may primarily participate in the initiation or early immune regulation of COPD rather than in disease progression (Figures 4U–W). In summary, TIMP1, CLEC5A, PROK2, and CXCL8 demonstrated expression patterns closely associated with COPD severity, supporting their potential utility as biomarkers for disease staging and progression monitoring.

### 3.7 Immune microenvironment characteristics of COPD patients

**Figure 5:**
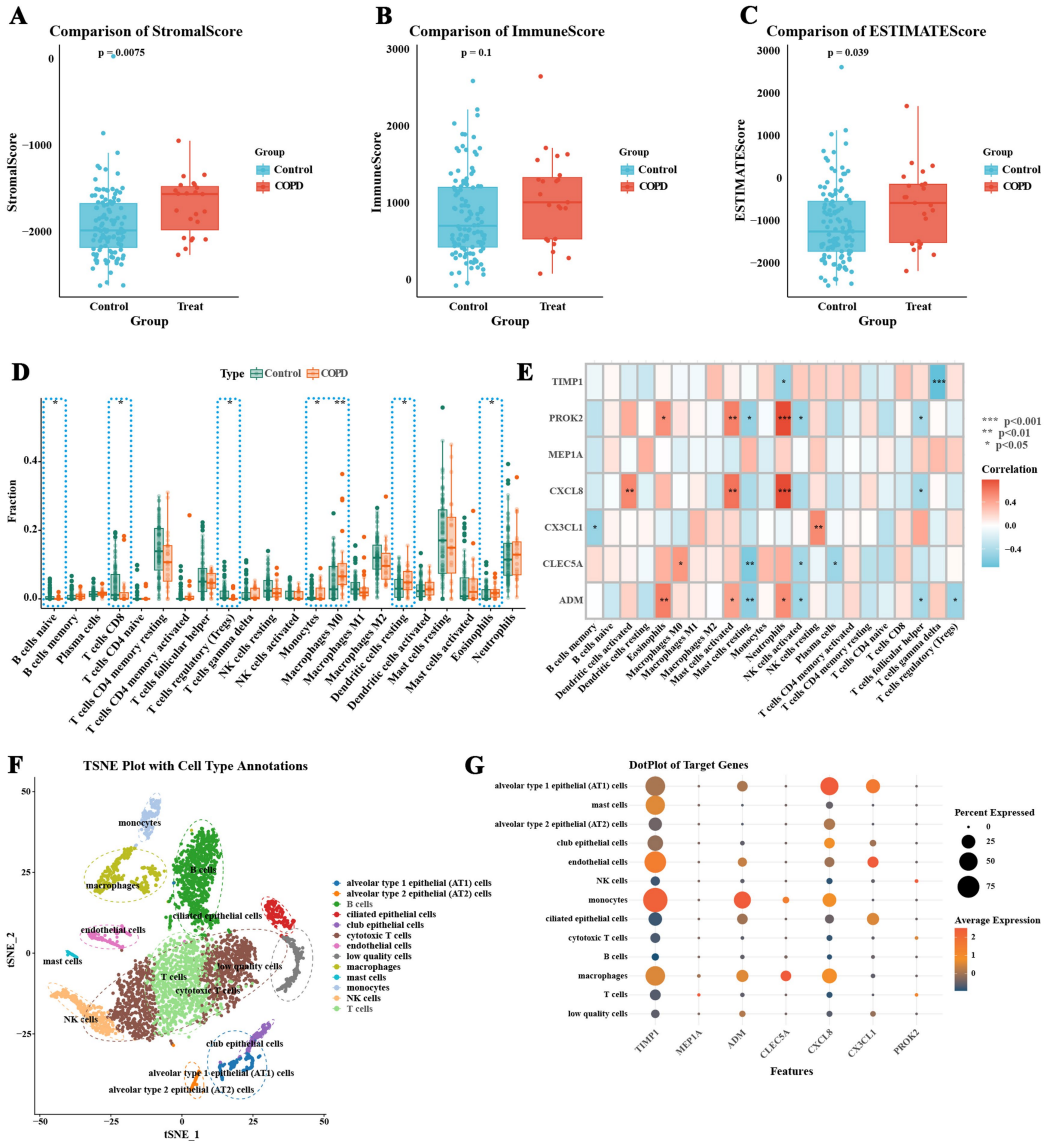
Immune microenvironment characteristics of COPD patients. **(A)** Comparison of ESTIMATE stromal scores. **(B)** Comparison of ESTIMATE immune scores. **(C)** Comparison of total ESTIMATE scores, indicating a significant increase in the COPD group. **(D)** Analysis of the relative proportions of 22 immune cell types in each sample using the CIBERSORT algorithm, comparing immune cell infiltration between the COPD group and the normal control group. **(E)** Correlation between the expression levels of COPD inflammatory feature genes and the infiltration proportions of various immune cells. **(F)** t-SNE plot of single-cell sequencing data, showing the distribution of different cell types. **(G)** Expression characteristics of COPD inflammatory feature genes at the single-cell level (* *p* < 0.05, ** *p* < 0.01, *** *p* < 0.001).

This study thoroughly examined the differences in the immune microenvironment of airway epithelial tissues between COPD patients and healthy individuals using the ESTIMATE algorithm, the CIBERSORT method. The results from the ESTIMATE algorithm showed that stromal scores were significantly higher in the COPD group compared to the normal group (Figure 5A, *p*-value = 0.0075; Supplementary Table 7), while no significant difference was observed in immune scores between the two groups (Figure 5B, *p*-value = 0.1). However, the total ESTIMATE scores were significantly higher in the COPD group (Figure 5C, *p*-value = 0.039), indicating that both stromal cell content and the overall immune microenvironment were notably altered in COPD patients. In addition, CIBERSORT analysis of the relative proportions of 22 immune cell types in each sample revealed notable differences between the COPD and normal groups (Figure 5D; Supplementary Table 8). The COPD group exhibited increased proportions of monocytes, M0 macrophages, eosinophils, and resting dendritic cells, while the normal group had a higher proportion of regulatory T cells (Tregs), CD8 + T cells, and naive B cells (Figure 5D). Correlation analysis further demonstrated that the infiltration levels of neutrophils and activated mast cells were significantly positively correlated with the expression levels of ADM, CXCL8, and PROK2. In addition, eosinophil infiltration was positively correlated with ADM and PROK2 expression, while activated dendritic cell infiltration showed a significant positive correlation with CXCL8 expression. Conversely, the infiltration of Tregs was negatively correlated with ADM expression (Figure 5E). These findings suggest that ADM, CXCL8, and PROK2 may play crucial roles in modulating immune cell infiltration and are central to the inflammatory pathology of COPD. Further single-cell sequencing data analysis revealed the expression characteristics of inflammatory feature genes in COPD at the single-cell level. Single-cell sequencing analysis of whole lung tissue samples (GSM5100998) from COPD patients in the GSE167295 dataset identified multiple cell subsets and annotated cell types (Figure 5F). The results show that monocytes express mainly TIMP1, ADM, and CXCL8. TIMP1 is also expressed in endothelial cells, macrophages, and mast cells (Figure 5G).

### 3.8 Pathway and immune characteristics of different inflammatory subtypes of COPD patients

**Figure 6:**
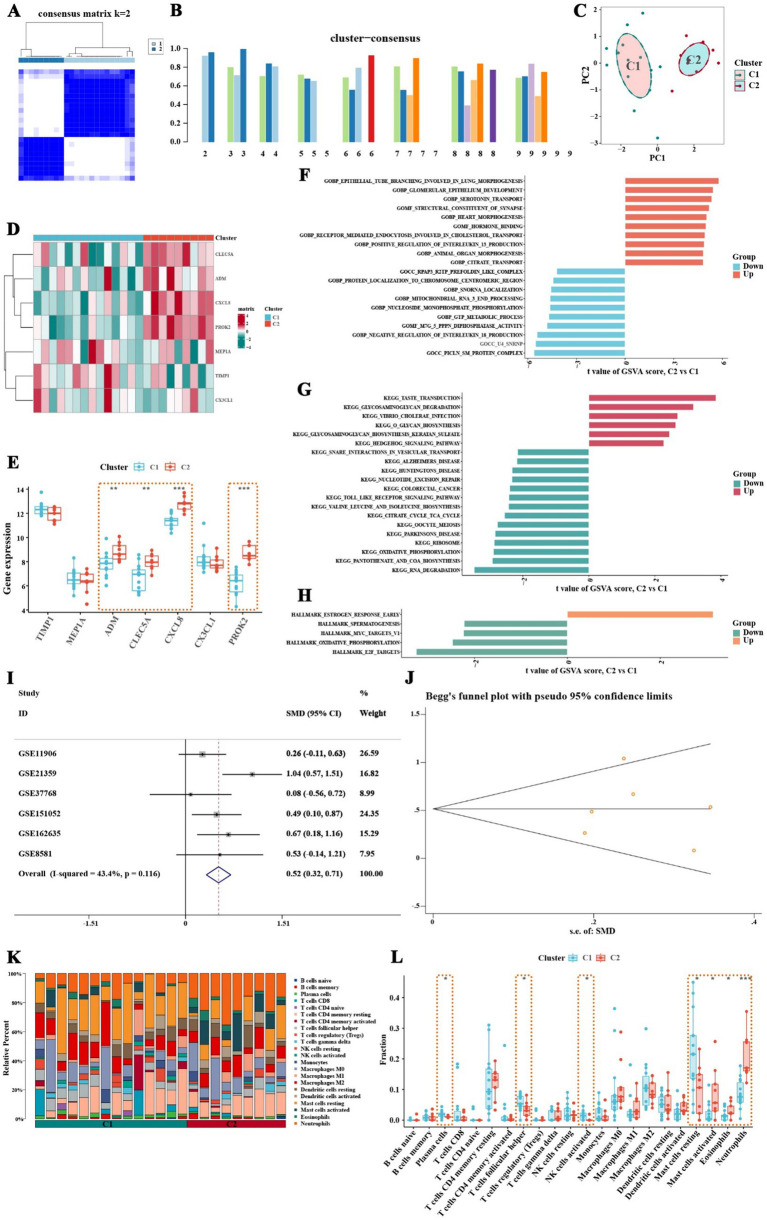
Classification of inflammatory subtypes, pathway activity, and immune characteristics in COPD patients, with CXCL8 expression validation. **(A)** Consistency matrix shows that when the k value is set to 2, the clustering effect of COPD patients is the best. **(B)** Consistency scores at different k values. **(C)** Principal component analysis shows clear separation between the two inflammatory subtypes of COPD patients. **(D,E)** Expression of inflammatory feature genes between different inflammatory subtypes of COPD patients. **(F)** GSVA analysis based on the c5.go.symbols.gmt gene set. **(G)** GSVA analysis based on the c2.cp.kegg.symbols.gmt gene set. **(H)** GSVA analysis based on the hall.v2022.1.Hs.symbols.gmt gene set. **(I)** Validation of CXCL8 expression using six datasets from the GEO database. **(J)** Publication bias test for the six datasets showed no significant bias. **(K,L)** Results of immune infiltration analysis between two inflammatory subtypes of COPD patients. Neutrophils, eosinophils, and activated mast cells are significantly increased in the C2 subtype, while this subtype also shows a trend of increased activated dendritic cells and M1 macrophages (* *p* < 0.05, ** *p* < 0.01, *** *p* < 0.001).

This study employed the ConsensusClusterPlus algorithm to classify COPD patients into distinct inflammatory subtypes. To further characterize these subtypes, GSVA and immune infiltration analysis were performed, focusing on differences in pathway activity and immune features among the subtypes. The clustering analysis identified the optimal number of clusters as 2 (Figure 6A). Consistency scores were calculated at different k values to assess the stability of the clusters (Figure 6B). The analysis revealed two distinct inflammatory subtypes in COPD patients, labeled as C1 and C2, which were clearly separable in principal component analysis (Figure 6C). Notably, significant differences in the expression of inflammation-related feature genes were observed between these two subtypes (Figure 6D). The C2 subtype exhibited significantly higher expression of genes such as CLEC5A, CXCL8, PROK2, and ADM (Figure 6E), with CXCL8 showing the highest expression in the C2 subtype. To further validate the expression of CXCL8, this study obtained five external datasets from the GEO database, including GSE11906, GSE37768, GSE151052, GSE162635, and GSE8581. Based on the expression data of 401 healthy control samples and 265 COPD samples from the external datasets, the standardized mean difference of CXCL8 indicated that CXCL8 was significantly upregulated in COPD samples (Figure 6I). The publication bias test indicated no publication bias in the six datasets, with a z-value of 0.56 and a corresponding *p*-value of 0.573 from Begg’s test (Figure 6J).

GSVA analysis further revealed differences in pathway activity between the inflammatory subtypes. Using the c5.go.symbols.gmt gene set, it was found that pathways promoting interleukin-13 production were significantly upregulated in the C2 subtype, whereas pathways that inhibit interleukin-18 production were suppressed in this group (Figure 6F; Supplementary Table 9). Additionally, analysis with the c2.cp.kegg.symbols.gmt gene set revealed that glycosaminoglycan degradation pathways were upregulated in the C2 subtype, while Toll-like receptor signaling pathways were significantly downregulated (Figure 6G; Supplementary Table 10). Results from the hall.v2022.1.Hs.symbols.gmt gene set indicated that early estrogen response pathways were upregulated in the C2 subtype, whereas pathways related to spermatogenesis, MYC targets V1, oxidative phosphorylation, and E2F targets were notably downregulated (Figure 6H; Supplementary Table 11). Immune infiltration analysis demonstrated a significant increase in neutrophil proportions in the C2 subtype (Figure 6K). Box plots confirmed that neutrophils, eosinophils, and activated mast cells were significantly elevated in the C2 subtype, and there was also a trend of increased infiltration of activated dendritic cells and M1 macrophages in this group (Figure 6L). These findings suggest that the C2 inflammatory subtype is characterized by distinct immune and pathway activity changes, which may have implications for understanding the immune landscape and pathology of COPD.

### 3.9 Potential of cinnamaldehyde targeting CXCL8 and assessment of the dynamics stability of the protein complex

**Figure 7:**
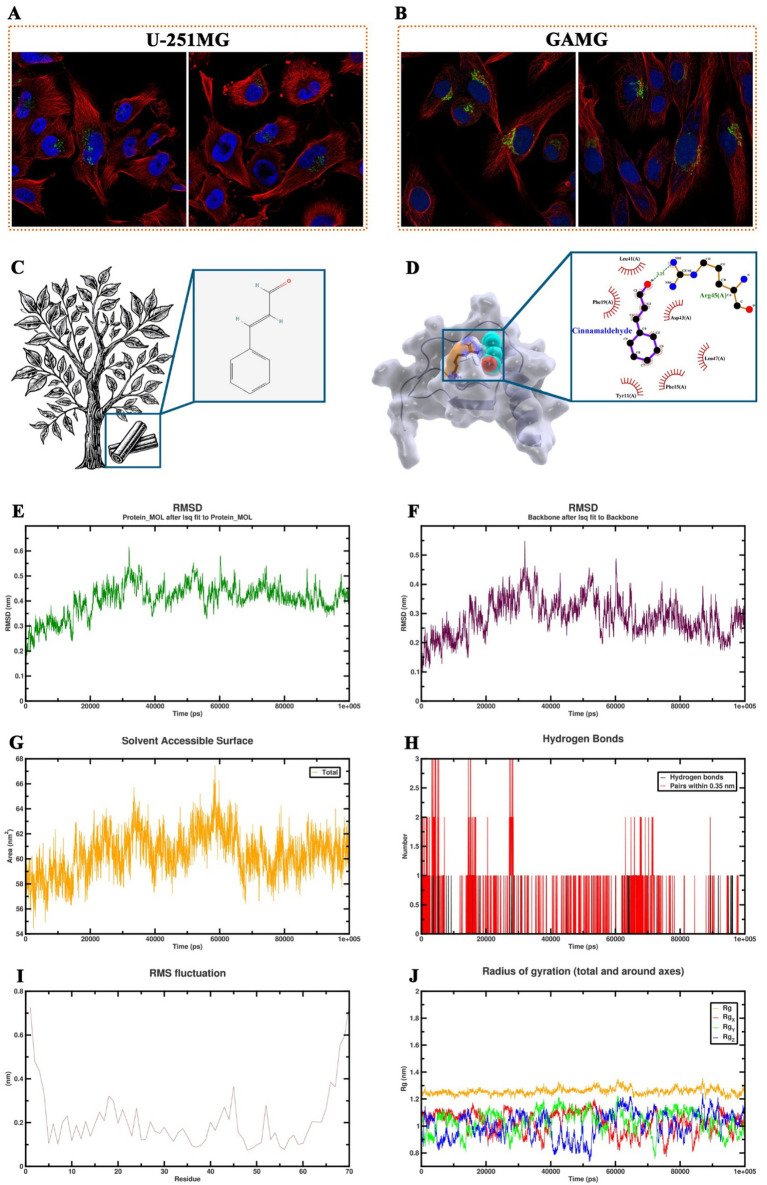
Molecular dynamics simulation and binding analysis of cinnamaldehyde with CXCL8. **(A,B)** Immunofluorescence images from the human protein atlas database showing that CXCL8 is predominantly localized in the Golgi apparatus of U-251MG and GAMG cell lines. **(C)** Molecular structure of cinnamaldehyde. **(D)** Molecular docking simulation showing the binding of cinnamaldehyde to the active pocket of CXCL8, with hydrogen bonding to Arg45 and hydrophobic interactions with Leu41, Asp43, Phe15, and other residues. **(E,F)** Root mean square deviation change trend of the cinnamaldehyde-CXCL8 complex and the protein backbone during the 100 ns molecular dynamics simulation. **(G)** Solvent accessible surface area change trend over time for the cinnamaldehyde-CXCL8 complex. **(H)** Number and time distribution of hydrogen bonds formed between cinnamaldehyde and CXCL8 during the binding process. **(I)** Root mean square fluctuation analysis at the residue level for the cinnamaldehyde-CXCL8 complex. **(J)** Radius of gyration changes in each axis direction and overall for the cinnamaldehyde-CXCL8 complex.

To further explore the mechanisms underlying the role of TCM active ingredients in the regulation of COPD-associated inflammation, this study screened the HERB database and identified cinnamaldehyde as a potential target for the key inflammatory factor CXCL8. Cinnamaldehyde, primarily derived from the *Cinnamomum* genus, has the molecular structure shown in Figure 7C. Immunofluorescence imaging from the Human Protein Atlas database revealed that CXCL8 is predominantly localized in the Golgi apparatus within cells, with consistent distribution observed in both U-251MG and GAMG cell lines (Figures 7A,B). Molecular docking simulations indicated that cinnamaldehyde stably binds to the active pocket of CXCL8, forming a hydrogen bond with Arg45 and hydrophobic interactions with Leu41, Asp43, Phe15, and other residues (Figure 7D). The binding energy of −5.2 kcal/mol suggests strong binding affinity (Table 2). To further validate the stability of this complex under physiological conditions, we conducted a 100 ns molecular dynamics simulation of the cinnamaldehyde-CXCL8 complex using GROMACS. The system was parameterized with the CHARMM36 force field and the TIP3P water model, followed by energy minimization and NVT/NPT equilibration. RMSD analysis revealed that after initial fluctuations, the complex stabilized (Figures 7E,F), indicating that the overall structure remained stable. SASA analysis showed stable fluctuations in the solvent-accessible surface area (Figure 7G), suggesting no significant conformational collapse. Hydrogen bond analysis revealed that cinnamaldehyde formed stable hydrogen bond interactions with CXCL8 throughout the binding process (Figure 7H). RMSF results indicated minimal fluctuations in the key binding residues of the complex (Figure 7I), and Rg analysis supported the good structural stability of the complex (Figure 7J). In conclusion, cinnamaldehyde can stably bind and target CXCL8, with minimal conformational fluctuations during binding, indicating its potential value in the development of drugs targeting COPD-related inflammatory responses.

**Table 2:** Summary of molecular docking results of cinnamaldehyde and CXCL8 protein.

Mode	Affinity (kcal/mol)	RMSD l.b.	RMSD u.b.
1	−5.4	0.000	0.000
2	−5.2	3.803	5.490
3	−5.1	2.543	3.179
4	−5.1	3.248	4.677
5	−4.6	4.304	5.570
6	−4.4	12.261	13.877
7	−4.4	2.917	3.495
8	−4.3	3.090	3.843
9	−4.2	8.893	9.552
10	−3.8	17.530	18.922

## 4 Discussion

COPD is characterized by airflow limitation and chronic inflammation, with the inflammatory response playing a crucial role in the onset and progression of the disease (29). Extensive studies have demonstrated that COPD patients exhibit persistent inflammation in the airways and lung tissues, primarily characterized by infiltration of neutrophils, macrophages, and T lymphocytes (30). Inflammatory factors such as TNF-*α*, IL-6, IL-8, and chemokines play a significant role in the pathogenesis of COPD by promoting airway remodeling and structural damage (20, 31). Although the inflammation in COPD can be alleviated by regulating these inflammatory factors, current therapeutic strategies remain limited (32). Therefore, exploring the genes associated with inflammation in COPD, particularly key genes involved in inflammation, can provide new insights for early diagnosis and targeted therapy.

In this study, a differential gene expression analysis of small airway epithelial tissues from COPD patients and healthy controls were conducted. And we identified 495 differentially expressed genes, many of which are related to inflammation. GO and KEGG enrichment analyses revealed that these differentially expressed genes play important roles in inflammation-related pathways such as the response to exogenous stimuli, arachidonic acid metabolism, and the IL-17 signaling pathway. Further GSEA indicated that inflammation and TNF-α signaling pathways were significantly activated in the epithelial tissues of COPD patients. By cross-referencing inflammation-related genes from the literature with the differentially expressed genes, we identified 14 key inflammatory genes that confirm the ongoing immune response and tissue damage in COPD. Feature selection using Lasso regression and random forest models identified genes such as TIMP1, CLEC5A, and CXCL8, which exhibited high diagnostic value, showing strong sensitivity and specificity in ROC curve analysis. These findings provide potential genetic biomarkers for the early diagnosis and targeted treatment of COPD.

The pathogenesis of COPD is characterized by airway remodeling and persistent inflammation in the small airways, which are closely associated with several biological processes and signaling pathways identified in this study (30, 33–35). Specifically, the processes of keratinocyte differentiation, epidermal cell differentiation, and response to xenobiotic stimulus were found to be significantly enriched in COPD small airway epithelial tissues. The dysregulation of keratinocyte and epidermal cell differentiation processes may be associated with airway epithelial cell remodeling and abnormal proliferation in COPD patients, which may compromise the airways’ ability to respond to environmental stimuli, exacerbating disease progression (36). Additionally, the enrichment of pathways like arachidonic acid metabolism and hormone metabolism suggests their involvement in COPD pathophysiology. Arachidonic acid metabolites, particularly leukotrienes and prostaglandins, are known to play pivotal roles in the inflammatory response in COPD (37, 38). By activating their receptors, these metabolites initiate inflammatory cascades, leading to the recruitment of immune cells and further airway damage (39). This finding corroborates previous studies that have highlighted the importance of arachidonic acid metabolism in COPD exacerbations (40). The analysis also revealed significant enrichment of the IL-17 and TNF-*α* signaling pathways, both of which are central to the inflammatory response in COPD. The IL-17 signaling pathway and TNF signaling pathway play key roles in inflammatory responses. The former promotes neutrophil recruitment and activation, thus playing an important role in chronic inflammation in COPD (41). Studies have shown that IL-17 can induce epithelial cells and fibroblasts to produce various chemokines and cytokines, enhancing inflammatory responses and tissue destruction (42). TNF-α is an important inflammatory mediator in COPD, with elevated levels closely associated with disease severity and lung function decline (43). Cell adhesion molecules in COPD may participate in inflammatory responses and tissue remodeling by regulating leukocyte adhesion and migration (44–46). Furthermore, the role of retinoic acid metabolism and cytochrome P450 enzymes in COPD was underscored. Retinoic acid, a metabolite of retinol, plays an important role in maintaining epithelial cell integrity and immune function, and its metabolic disorder may exacerbate the condition of COPD patients (47). Cytochrome P450 enzymes play key roles in drug metabolism and oxidative stress response, and their activity alterations may affect the response to drugs and oxidative stress levels of COPD patients (48–50). Furthermore, CYP450 gene polymorphisms are associated with COPD susceptibility. CYP2J2 is an important member of the cytochrome P450 family, playing a key role in the metabolism of arachidonic acid (51). Studies have confirmed that CYP2J2 gene polymorphisms are significantly associated with COPD susceptibility in the Chinese Han population (52). The GSEA analysis further revealed significant activation of pathways such as inflammatory response and TNF-α signaling via NF-κB, which supports the notion of chronic inflammation driving COPD progression. NF-κB activation can induce the expression of various cytokines, chemokines, and adhesion molecules, promoting the recruitment and activation of inflammatory cells, thus forming a chronic inflammatory response (53). Existing studies have shown that NF-κB signaling participates in airway inflammatory responses in patients with exacerbated COPD by regulating the expression of various inflammatory mediators (54). The significant activation of these pathways further reveals the presence of intense inflammatory responses and abnormal cell signaling in COPD patients, supporting the key role of inflammation in the development and progression of COPD.

In addition to inflammation-related pathways, this study also identified several important pathways closely associated with metabolic regulation, cellular function maintenance, and endocrine modulation, further revealing the complex, multi-factorial, and multi-system interactions underlying the pathogenesis of COPD. Houssaini et al. demonstrated that the mTOR signaling pathway is significantly activated in the lung tissue of COPD patients (55). Activation of the mTOR pathway induces senescence in pulmonary arterial smooth muscle cells and endothelial cells, inhibits the expression of autophagy-related proteins such as LC3, ATG3, and ATG5, and promotes the release of inflammatory cytokines IL-6, IL-8, and CCL2, driving typical pathological changes of COPD, including emphysema, pulmonary arterial hypertension, and chronic inflammation (55). Pathways related to structural remodeling were also widely identified in the enrichment analysis of this study. GSEA further revealed significant activation of the EMT pathway in the COPD group. EMT is the process by which epithelial cells lose polarity and acquire mesenchymal characteristics, and it is closely associated with pulmonary fibrosis, airway narrowing, and gas exchange abnormalities (56, 57). Previous studies have shown that repeated epithelial damage and chronic inflammation in COPD can induce EMT activation, leading to basement membrane destruction and stromal deposition (58). Abnormal activation of the KRAS signaling pathway may drive the abnormal proliferation of airway epithelial cells and airway remodeling, further compromising the structural stability of small airways (59). O-glycosylation plays an important role in regulating the synthesis and secretion of airway mucus proteins, affecting mucus viscosity and clearance efficiency (60). In this study, the enrichment of the mucin-type O-glycosylation pathway suggests that abnormalities in the glycosylation of mucus molecules may increase secretion viscosity and obstruct clearance, thus exacerbating the risk of infection and airway obstruction (61, 62). Furthermore, dysregulation of fatty acid metabolism was significantly enriched in COPD in this study, possibly related to lipid peroxidation and its associated pro-apoptotic processes. Previous research has indicated that lipid peroxidation generates reactive aldehydes and oxidized sterols, leading to cell membrane damage, mitochondrial dysfunction, and inflammatory responses, thereby contributing to airway damage in COPD (63–65). In terms of immune-endocrine regulation, estrogen affects both innate and adaptive immune responses, and it’s signaling imbalance may lead to abnormal immune cell activity, triggering enhanced inflammation or immune dysfunction (66). This study found that both early and late estrogen response pathways were upregulated in COPD patients. Abnormal activation of estrogen and its receptor signaling may exacerbate neutrophil-mediated inflammatory responses, thereby aggravating the pathological progression of COPD (67, 68). In conclusion, beyond inflammation, mechanisms such as metabolic dysregulation, mucus dysfunction, abnormal cell autophagy and apoptosis, and endocrine imbalance are deeply involved in the pathological process of COPD, providing new theoretical insights for understanding its complex pathogenesis and formulating more precise intervention strategies.

Subsequently, this study screened 14 inflammation-related COPD genes, which may play important roles in the inflammatory response of COPD. Lasso regression and random forest algorithms further screened the following seven feature genes for COPD inflammatory response: TIMP1, MEP1A, ADM, CLEC5A, CXCL8, CX3CL1, and PROK2. CX3CL1 was downregulated in COPD patients, while the other six genes were upregulated. The COPD risk prediction model constructed with these seven inflammation-related COPD feature genes showed good accuracy and clinical application value. Upregulation of the expression of inflammatory feature genes plays an important role in the inflammatory response and airway remodeling of COPD. Studies have shown that, as a metalloproteinase, MEP1A participates in tissue remodeling and cell migration by degrading various matrix proteins and cell surface molecules (69–71). Additionally, MEP1A can regulate the secretion of inflammatory mediators, affecting inflammatory responses. Inhibition of MEP1A expression can downregulate the secretion of the pro-inflammatory mediator IL-6 (72). CLEC5A is related to the activation of immune cells and inflammatory responses (73). As a pattern recognition receptor, CLEC5A can recognize pathogen-associated molecular patterns and activate immune responses (74, 75). Studies have shown that CLEC5A is highly expressed in various inflammatory diseases, and its inhibition can alleviate inflammatory responses and tissue damage (75, 76). In COPD, high expression of CLEC5A may promote chronic inflammation by enhancing macrophage activation and inflammatory mediator release (74, 77). PROK2 is involved in COPD by regulating inflammatory responses and cell apoptosis (78, 79). By binding to its G-protein-coupled receptors PKR1 and PKR2, PROK2 promotes chemotaxis and the release of pro-inflammatory cytokines, thus exacerbating the inflammatory response and tissue damage in COPD (80–82). CXCL8 is a potent neutrophil chemokine (83, 84). In COPD, high expression of CXCL8 is closely related to increased neutrophils in the airways (22). By binding to its receptors CXCR1 and CXCR2, CXCL8 induces neutrophil migration, degranulation, and oxidative burst, thus enhancing the inflammatory response (85, 86). This may be an important mechanism for airway inflammation and damage in COPD patients. Studies have shown that inhibition of the CXCL8 signaling pathway can alleviate airway inflammation and functional impairment in COPD patients (87). Therefore, inhibition of the CXCL8 signaling pathway may become a new strategy for COPD treatment.

Immune cell infiltration analysis showed significant changes in the distribution of various immune cells in the small airway epithelial tissues of COPD patients. Monocytes, M0 macrophages, eosinophils, and resting dendritic cells were significantly increased in COPD patients, while Tregs, CD8 + T cells, and naive B cells were lower than those in the normal group. Single-cell sequencing data further revealed that CXCL8 is mainly expressed in endothelial cells, macrophages, and monocytes. Neutrophils promote the inflammatory response and tissue damage in COPD by releasing various inflammatory mediators and proteases (13). The role of eosinophils in the development of COPD may be related to their role in allergic inflammation and airway remodeling (88, 89). Activation of mast cells promotes airway inflammation and hyperreactivity in COPD by releasing histamine and other inflammatory mediators (90). Tregs play key roles in maintaining immune tolerance and inhibiting excessive inflammatory responses. Their reduction may lead to immune response dysregulation in COPD patients, further exacerbating the inflammatory response (91–93).

Subsequently, based on the above inflammatory feature genes, this study classified COPD patients into two inflammatory subtypes. Inflammatory-related genes such as CLEC5A, CXCL8, and PROK2 were significantly upregulated in the C2 subtype. GSVA analysis demonstrated pathway activity differences between the two inflammatory subtypes of COPD patients. Pathways regulating interleukin-13 and interleukin-18 production were significantly activated in the C2 subtype. IL-13 is a key Th2 cytokine that promotes airway remodeling and mucus secretion, playing an important role in chronic inflammation in COPD (94, 95). Plasma IL-13 levels are significantly elevated in COPD patients (96). Additionally, IL-13 is associated with an increased risk of developing COPD. Studies have confirmed that IL-13 gene polymorphisms (such as rs20541 and rs1800925) are associated with an increased risk of COPD in the southern Chinese Han population (96). As a pro-inflammatory and pro-apoptotic cytokine, IL-18 is expressed primarily in alveolar macrophages and bronchial and alveolar epithelial cells, promoting airway obstruction and inflammatory responses by activating and migrating inflammatory cells (97, 98). Studies have confirmed that serum IL-18 levels are significantly elevated in COPD patients, especially during acute exacerbations (98, 99). Furthermore, IL-18 levels are negatively correlated with lung function decline (99). However, the functional differences between the C1 and C2 subtypes remain to be fully explored. The C1 subtype, which exhibited lower expression of pro-inflammatory cytokines like IL-13 and IL-18, may represent a less inflammatory phenotype, potentially linked to a milder progression of COPD. In contrast, the C2 subtype, characterized by a more pronounced inflammatory profile, likely reflects a more severe and progressive form of COPD. These functional distinctions between the two subtypes may influence not only the progression of the disease but also the response to therapy. The upregulation of pathways associated with IL-13 and IL-18 in the C2 subtype suggests a Th2-skewed immune response, which could have implications for targeted therapies, such as IL-13 antagonists, in patients with this subtype (100). Moreover, the immune infiltration analysis revealed significant changes in immune cell distributions, with the C2 subtype showing a marked increase in neutrophil and eosinophil infiltration, both of which are known to contribute to airway remodeling and tissue damage. These findings highlight that the functional differences between C1 and C2 may not only reflect variations in inflammatory gene expression but also in immune cell interactions and responses, potentially guiding the development of subtype-specific therapeutic strategies. Thus, further functional studies are needed to explore the underlying mechanisms that differentiate the inflammatory profiles of C1 and C2 subtypes, including detailed analyses of immune cell activation and cytokine production. Understanding these differences will be crucial in determining whether specific inflammatory subtypes respond better to certain therapies and may also reveal novel biomarkers for disease severity and progression.

Finally, this study screened and validated the potential therapeutic effects of cinnamaldehyde, a TCM monomer component, particularly its targeting effects on the CXCL8 protein. Cinnamaldehyde, a natural compound derived from cinnamon bark, has been extensively studied for its anti-inflammatory, antioxidant, and immunomodulatory properties. It has shown significant therapeutic potential in treating various inflammatory diseases by reducing the levels of reactive oxygen species, nitric oxide, TNF-*α*, IL-6, and IL-10 (101–104). In addition to its antioxidant effects, cinnamaldehyde is known to suppress the activation of the NF-κB pathway, thereby reducing the expression of pro-inflammatory cytokines and chemokines (105). It also modulates the MAPK signaling pathway, involved in cellular responses to stress, and inhibits the production of pro-inflammatory mediators such as cyclooxygenase-2 (106, 107). Furthermore, cinnamaldehyde has been shown to inhibit inducible nitric oxide synthase expression, further mitigating nitric oxide-mediated inflammation (107, 108). Through molecular docking simulations, this study found that cinnamaldehyde could bind to the active site of the CXCL8 protein, suggesting that it may exert its anti-inflammatory effects by regulating the function of CXCL8. CXCL8 is a chemokine involved in neutrophil recruitment and activation during the inflammatory response. And by targeting it, cinnamaldehyde may help reduce the recruitment of inflammatory cells, thereby ameliorating chronic inflammation in COPD. Taken together, these findings provide new insights into the potential of cinnamaldehyde as a therapeutic agent for inflammatory diseases like COPD. Its ability to modulate key inflammatory pathways and target critical proteins such as CXCL8 positions cinnamaldehyde as a promising candidate for personalized treatment strategies aimed at mitigating COPD symptoms and progression.

In conclusion, this study revealed significantly enriched biological processes and pathways in COPD patients using various analytical methods and screened multiple inflammation-related COPD feature genes. Immune infiltration analysis and single-cell sequencing data further explored the important roles of these genes in different types of immune cells. Additionally, this study identified two inflammatory subtypes of COPD based on inflammation-related COPD feature genes, and it analyzed differences in pathway activity in different inflammatory subtypes of COPD patients using GSVA. Finally, the potential therapeutic effects of cinnamaldehyde were screened and validated by molecular-protein docking. However, one limitation of this study is the lack of detailed clinical data regarding the different disease stages of COPD, particularly the acute exacerbation phase and stable phase. As COPD patients exhibit distinct cellular profiles and gene expression patterns between these stages. Further analysis of clinical data, especially focusing on disease staging, is necessary for a more comprehensive understanding. This study provides important theoretical support for research into the molecular mechanism and personalized treatment of COPD, but further experimental validation and clinical studies are needed to determine the specific roles and therapeutic potential of these genes and pathways in COPD. Future research should focus on functional validation of these feature genes, as well as the development of potential therapeutic targets, to provide more effective treatment strategies for COPD patients.

## Data Availability

The original contributions presented in the study are included in the article/Supplementary material, further inquiries can be directed to the corresponding author.
