# Supplementary material for: Analysis of immune characteristics and inflammatory mechanisms in COPD patients: a multi-layered study combining bulk and single-cell transcriptome analysis and machine learning
Source: Front Med (Lausanne). 2025 Jul 21;12:1592802. doi: 10.3389/fmed.2025.1592802 (PMC12318759; doi:10.3389/fmed.2025.1592802)
Supplement: Supplementary file 1 [file Table_1.docx]

**Supplementary Table 1. List of Differentially Expressed Genes in COPD Based on Analysis of the GSE21359 Dataset.**

| **gene_ID** | **logFC** | **AveExpr** | **t** | **P.Value** | **adj.P.Val** |
| --- | --- | --- | --- | --- | --- |
| OSBPL6 | -0.70869 | 10.82044 | -7.65289 | 3.27E-12 | 3.81E-08 |
| SPRR3 | 2.172583 | 5.723926 | 7.497156 | 7.60E-12 | 5.55E-08 |
| PROS1 | -1.16423 | 12.65507 | -7.45534 | 9.52E-12 | 5.55E-08 |
| KRT13 | 2.071522 | 4.739963 | 7.301125 | 2.18E-11 | 7.33E-08 |
| SCGB3A1 | -0.81221 | 15.25972 | -7.29745 | 2.22E-11 | 7.33E-08 |
| FAM3B | 1.029586 | 9.696044 | 7.264354 | 2.65E-11 | 7.33E-08 |
| TCN1 | 1.884781 | 8.431014 | 7.260354 | 2.70E-11 | 7.33E-08 |
| LOC283177 | -1.1863 | 7.847856 | -7.25188 | 2.83E-11 | 7.33E-08 |
| KRT6A | 2.247305 | 5.690833 | 6.967096 | 1.27E-10 | 2.72E-07 |
| DGKA | 0.691142 | 8.271636 | 6.965208 | 1.28E-10 | 2.72E-07 |
| C2orf70 | 0.86116 | 8.724874 | 6.799086 | 3.05E-10 | 5.58E-07 |
| FGFBP1 | 0.978814 | 8.50204 | 6.795306 | 3.11E-10 | 5.58E-07 |
| SFN | 0.88722 | 10.82159 | 6.710329 | 4.82E-10 | 8.03E-07 |
| LTF | -2.10209 | 10.52158 | -6.6174 | 7.76E-10 | 1.21E-06 |
| CEACAM5 | 2.178767 | 10.78894 | 6.571589 | 9.81E-10 | 1.43E-06 |
| TENM4 | 0.633454 | 8.308811 | 6.542632 | 1.14E-09 | 1.56E-06 |
| SRPX2 | 1.374472 | 7.334302 | 6.531227 | 1.20E-09 | 1.56E-06 |
| PHLDA1 | 0.799361 | 6.393678 | 6.392094 | 2.43E-09 | 2.84E-06 |
| TPRXL | 1.971339 | 6.698771 | 6.325627 | 3.40E-09 | 3.60E-06 |
| FHOD3 | -0.99776 | 9.362759 | -6.17261 | 7.26E-09 | 7.06E-06 |
| FAM110C | 0.877184 | 7.862604 | 6.135501 | 8.72E-09 | 8.14E-06 |
| AHRR | 1.59849 | 6.876887 | 6.127433 | 9.08E-09 | 8.14E-06 |
| CYP1A1 | 3.496824 | 6.303249 | 6.10487 | 1.01E-08 | 8.76E-06 |
| CYP1B1 | 3.416316 | 8.534753 | 6.091504 | 1.08E-08 | 8.77E-06 |
| KLHDC7A | -0.67536 | 8.569698 | -6.08997 | 1.09E-08 | 8.77E-06 |
| CLDN10 | 0.973938 | 11.4314 | 6.00997 | 1.61E-08 | 1.25E-05 |
| ATP10B | 0.73433 | 7.339148 | 5.988886 | 1.79E-08 | 1.30E-05 |
| GPT2 | 0.75703 | 7.481736 | 5.9837 | 1.83E-08 | 1.30E-05 |
| GAS6 | -0.5915 | 9.351546 | -5.97501 | 1.91E-08 | 1.31E-05 |
| SERTAD4-AS1 | -0.60118 | 9.907789 | -5.92603 | 2.42E-08 | 1.57E-05 |
| CXCL14 | 1.92666 | 5.128707 | 5.898796 | 2.76E-08 | 1.74E-05 |
| CLEC5A | 2.093424 | 5.465826 | 5.873016 | 3.13E-08 | 1.92E-05 |
| STEAP4 | -0.72642 | 11.02908 | -5.78948 | 4.67E-08 | 2.66E-05 |
| ANXA3 | 0.773755 | 10.08425 | 5.763143 | 5.30E-08 | 2.94E-05 |
| SLC51A | 0.709574 | 9.142494 | 5.743844 | 5.81E-08 | 3.08E-05 |
| CD109 | 0.86405 | 6.54987 | 5.740041 | 5.91E-08 | 3.08E-05 |
| STATH | 1.848693 | 4.721977 | 5.73453 | 6.07E-08 | 3.08E-05 |
| GMDS | 0.64551 | 9.157121 | 5.718989 | 6.53E-08 | 3.15E-05 |
| AFAP1L1 | -0.67317 | 7.349215 | -5.71767 | 6.57E-08 | 3.15E-05 |
| ADAMTS15 | -0.76011 | 6.366797 | -5.7121 | 6.75E-08 | 3.15E-05 |
| CYP4X1 | -0.76976 | 11.8552 | -5.71178 | 6.76E-08 | 3.15E-05 |
| MEP1A | 1.513385 | 5.248823 | 5.680346 | 7.84E-08 | 3.52E-05 |
| GALNT5 | 0.739943 | 7.851206 | 5.658282 | 8.70E-08 | 3.83E-05 |
| ADM | 1.204568 | 7.184908 | 5.594685 | 1.17E-07 | 4.89E-05 |
| TIMP1 | 0.745004 | 11.58756 | 5.56089 | 1.37E-07 | 5.62E-05 |
| TMEM45A | -1.28034 | 11.33592 | -5.54757 | 1.46E-07 | 5.88E-05 |
| SLIT2 | -1.05314 | 9.673916 | -5.44814 | 2.32E-07 | 8.19E-05 |
| TMEM200A | 1.018544 | 6.978268 | 5.438901 | 2.42E-07 | 8.42E-05 |
| ITGA6 | 0.737032 | 7.240069 | 5.43517 | 2.46E-07 | 8.44E-05 |
| FHOD1 | 0.625635 | 8.401795 | 5.422222 | 2.61E-07 | 8.83E-05 |
| SSFA2 | 0.600865 | 7.696322 | 5.410082 | 2.76E-07 | 9.20E-05 |
| RASSF10 | -0.6246 | 9.20223 | -5.4026 | 2.86E-07 | 9.39E-05 |
| DLL1 | -0.63309 | 9.163617 | -5.39624 | 2.94E-07 | 9.50E-05 |
| CEACAM6 | 0.649943 | 13.78445 | 5.392339 | 3.00E-07 | 9.50E-05 |
| B3GNT6 | 1.338763 | 6.344828 | 5.391059 | 3.01E-07 | 9.50E-05 |
| GAD1 | 1.297266 | 5.834718 | 5.37348 | 3.27E-07 | 9.97E-05 |
| WFDC6 | -0.78648 | 8.975134 | -5.37181 | 3.29E-07 | 9.97E-05 |
| MGP | -1.26443 | 8.365425 | -5.35734 | 3.52E-07 | 0.000105 |
| SPRR1B | 1.587778 | 5.343081 | 5.354176 | 3.57E-07 | 0.000105 |
| MTHFD2 | 0.783846 | 8.081696 | 5.329593 | 3.99E-07 | 0.000115 |
| UPK1B | 1.144205 | 10.08411 | 5.327297 | 4.03E-07 | 0.000115 |
| CCL2 | 2.015293 | 6.618963 | 5.318538 | 4.20E-07 | 0.000118 |
| TMEM178A | -0.76681 | 10.91201 | -5.30472 | 4.47E-07 | 0.000123 |
| CLDN8 | -0.86143 | 10.95342 | -5.29169 | 4.74E-07 | 0.000129 |
| PAX1 | -1.21796 | 6.069527 | -5.28182 | 4.96E-07 | 0.000133 |
| LOC284825 | 1.739677 | 7.419209 | 5.272651 | 5.17E-07 | 0.000135 |
| SLC29A1 | -0.88922 | 7.762668 | -5.25985 | 5.47E-07 | 0.000137 |
| LA16c-380H5.5 | -0.71684 | 7.983844 | -5.25516 | 5.59E-07 | 0.000138 |
| FBXL7 | 0.633147 | 8.183949 | 5.213795 | 6.74E-07 | 0.000163 |
| WFDC1 | 1.215625 | 6.882732 | 5.20144 | 7.12E-07 | 0.000169 |
| SGCE | -0.77603 | 8.717589 | -5.19101 | 7.46E-07 | 0.000176 |
| PYCR1 | 0.792111 | 8.10726 | 5.186955 | 7.60E-07 | 0.000177 |
| FOXA2 | -1.02595 | 7.62837 | -5.17408 | 8.05E-07 | 0.000185 |
| SLC2A3 | 1.22493 | 6.780511 | 5.161136 | 8.53E-07 | 0.000191 |
| PCDH17 | -1.1122 | 7.947354 | -5.14008 | 9.37E-07 | 0.000198 |
| DEFB1 | 1.042443 | 8.27165 | 5.139745 | 9.38E-07 | 0.000198 |
| PEG10 | -0.76139 | 7.520673 | -5.13862 | 9.43E-07 | 0.000198 |
| MMP12 | 1.535916 | 6.900638 | 5.136845 | 9.50E-07 | 0.000198 |
| SLC7A11 | 2.038791 | 7.972717 | 5.118384 | 1.03E-06 | 0.000211 |
| C3 | -1.33569 | 12.33265 | -5.06793 | 1.29E-06 | 0.000256 |
| CLIP4 | 0.812574 | 9.5674 | 5.045853 | 1.42E-06 | 0.000271 |
| LOC100506558 | 0.905256 | 6.099779 | 5.044906 | 1.43E-06 | 0.000271 |
| PTHLH | 1.103594 | 4.946843 | 5.023839 | 1.57E-06 | 0.000288 |
| ASTN2 | -0.60025 | 9.334012 | -5.02161 | 1.58E-06 | 0.000288 |
| SCEL | 1.28452 | 4.756427 | 5.015778 | 1.62E-06 | 0.000291 |
| NPAS3 | -0.70531 | 6.706972 | -5.01559 | 1.62E-06 | 0.000291 |
| PARM1 | 0.694829 | 7.82458 | 5.01222 | 1.65E-06 | 0.000292 |
| SLITRK6 | -0.77922 | 10.40715 | -4.98425 | 1.86E-06 | 0.000322 |
| CCDC108 | -1.09268 | 5.942309 | -4.97788 | 1.92E-06 | 0.000328 |
| CCDC81 | -0.62538 | 10.6695 | -4.94817 | 2.18E-06 | 0.000371 |
| WNK4 | -1.0361 | 6.561555 | -4.91767 | 2.49E-06 | 0.000412 |
| FCGR2B | 1.269551 | 6.421597 | 4.901948 | 2.66E-06 | 0.00043 |
| EFEMP1 | -0.59713 | 10.89626 | -4.87808 | 2.95E-06 | 0.000461 |
| RTN4RL1 | -0.94678 | 7.031943 | -4.87483 | 2.99E-06 | 0.000461 |
| SYTL4 | 0.784844 | 7.532785 | 4.874714 | 3.00E-06 | 0.000461 |
| SPP1 | 1.991076 | 8.357625 | 4.873657 | 3.01E-06 | 0.000461 |
| C14orf132 | -0.75216 | 10.11104 | -4.87096 | 3.05E-06 | 0.000461 |
| MUC12 | 1.178167 | 6.084552 | 4.849959 | 3.33E-06 | 0.000496 |
| UGT1A1 | 1.082544 | 8.949783 | 4.848591 | 3.35E-06 | 0.000496 |
| LINC00675 | 0.602268 | 8.050351 | 4.845842 | 3.39E-06 | 0.000496 |
| SCG3 | 1.595328 | 4.057744 | 4.833269 | 3.58E-06 | 0.000509 |
| GPX2 | 1.498234 | 9.8049 | 4.830848 | 3.62E-06 | 0.000511 |
| ASCL3 | 0.937494 | 7.93652 | 4.826643 | 3.68E-06 | 0.000516 |
| MLKL | 0.752024 | 7.932444 | 4.826128 | 3.69E-06 | 0.000516 |
| TRIM7 | 0.954309 | 7.000373 | 4.823788 | 3.73E-06 | 0.000518 |
| FRMD4A | -0.62464 | 7.333188 | -4.82214 | 3.76E-06 | 0.000518 |
| KCNA1 | -0.95366 | 7.572732 | -4.81122 | 3.93E-06 | 0.00054 |
| DUOX2 | 1.436726 | 7.221275 | 4.800156 | 4.13E-06 | 0.000556 |
| ITM2A | -0.96415 | 8.703696 | -4.79745 | 4.17E-06 | 0.000556 |
| IL19 | 1.062992 | 6.084721 | 4.791318 | 4.28E-06 | 0.000568 |
| PLA2G4A | 0.794914 | 7.97504 | 4.784774 | 4.40E-06 | 0.000576 |
| AKR1B10 | 2.673551 | 9.919565 | 4.780476 | 4.49E-06 | 0.000578 |
| ASRGL1 | 0.962568 | 7.616536 | 4.772618 | 4.64E-06 | 0.000588 |
| PLAT | 0.918545 | 7.086363 | 4.759552 | 4.90E-06 | 0.000615 |
| EPB41L2 | -0.69474 | 7.355069 | -4.7357 | 5.42E-06 | 0.000669 |
| CDC42EP5 | 0.788618 | 10.69097 | 4.73549 | 5.43E-06 | 0.000669 |
| ADD3-AS1 | 0.812745 | 5.884153 | 4.734623 | 5.45E-06 | 0.000669 |
| RP11-486A14.1 | 0.72383 | 8.156804 | 4.732345 | 5.50E-06 | 0.000672 |
| CABYR | 1.530058 | 8.04403 | 4.730445 | 5.55E-06 | 0.000674 |
| HS3ST3A1 | 1.623646 | 4.821319 | 4.721578 | 5.76E-06 | 0.000686 |
| ARHGAP28 | -0.93471 | 4.472282 | -4.71934 | 5.81E-06 | 0.000688 |
| CSTA | 0.724707 | 11.03974 | 4.708378 | 6.09E-06 | 0.000713 |
| PRKAR2B | -0.68734 | 9.062703 | -4.70286 | 6.23E-06 | 0.000727 |
| DPYSL3 | 0.993105 | 6.810272 | 4.687861 | 6.64E-06 | 0.00077 |
| RP11-157B13.7 | -0.9633 | 4.947783 | -4.67273 | 7.07E-06 | 0.000809 |
| SRXN1 | 0.752147 | 9.703309 | 4.65637 | 7.57E-06 | 0.000853 |
| NQO1 | 0.995408 | 13.13449 | 4.643365 | 8.00E-06 | 0.000886 |
| UCHL1 | 2.048837 | 8.347489 | 4.637438 | 8.20E-06 | 0.000899 |
| PTGS2 | 1.014128 | 6.401323 | 4.610943 | 9.15E-06 | 0.000986 |
| CPE | 1.056896 | 7.153692 | 4.610312 | 9.18E-06 | 0.000986 |
| HIBADH | -0.63467 | 9.491876 | -4.60935 | 9.21E-06 | 0.000986 |
| BLK | -0.82741 | 5.007521 | -4.58305 | 1.03E-05 | 0.001065 |
| IL1R2 | 1.253087 | 6.368791 | 4.57798 | 1.05E-05 | 0.001078 |
| LOC100130744 | -1.37258 | 4.550891 | -4.55357 | 1.16E-05 | 0.001183 |
| ST3GAL4-AS1 | 1.337478 | 6.518959 | 4.553342 | 1.16E-05 | 0.001183 |
| RP11-362K14.7 | 0.787593 | 8.444847 | 4.545887 | 1.20E-05 | 0.001204 |
| RP4-665N4.4 | -0.70379 | 7.401894 | -4.54485 | 1.20E-05 | 0.001204 |
| CXCL8 | 0.8992 | 11.13733 | 4.537568 | 1.24E-05 | 0.001236 |
| RP3-412A9.16 | -0.8775 | 5.462235 | -4.53446 | 1.26E-05 | 0.001246 |
| SLC2A14 | 0.765135 | 7.039611 | 4.512119 | 1.38E-05 | 0.001341 |
| CHDH | 0.62768 | 6.055666 | 4.508667 | 1.40E-05 | 0.001346 |
| PTGFR | -0.70459 | 9.85968 | -4.4722 | 1.62E-05 | 0.001508 |
| LHX6 | 0.680425 | 6.65305 | 4.456099 | 1.73E-05 | 0.00157 |
| ZNF467 | 0.594 | 6.963161 | 4.449696 | 1.78E-05 | 0.001599 |
| SEC14L3 | -1.54408 | 9.192452 | -4.43735 | 1.87E-05 | 0.001667 |
| GCNT3 | 0.676422 | 7.672368 | 4.436679 | 1.87E-05 | 0.001667 |
| PTPRH | 1.285111 | 5.852486 | 4.435339 | 1.88E-05 | 0.001669 |
| MUCL1 | 1.563902 | 11.16247 | 4.43109 | 1.92E-05 | 0.001685 |
| C1QTNF9B-AS1 | 0.769154 | 7.212297 | 4.426879 | 1.95E-05 | 0.001708 |
| PMAIP1 | 0.768652 | 7.895409 | 4.422525 | 1.98E-05 | 0.001719 |
| PLA2G7 | 1.292432 | 6.535195 | 4.418856 | 2.01E-05 | 0.00172 |
| IL1B | 0.976497 | 8.321636 | 4.415035 | 2.04E-05 | 0.001738 |
| FOLR1 | -0.6623 | 11.74502 | -4.41427 | 2.05E-05 | 0.001738 |
| GALNT6 | 0.676561 | 10.96112 | 4.402563 | 2.15E-05 | 0.001803 |
| ELMOD1 | 1.126931 | 7.444967 | 4.364695 | 2.50E-05 | 0.002034 |
| LIPF | -0.94942 | 5.172117 | -4.35304 | 2.62E-05 | 0.002116 |
| S100A10 | 0.662745 | 11.71652 | 4.349176 | 2.66E-05 | 0.002142 |
| MUC5B | -0.92186 | 10.80103 | -4.34695 | 2.69E-05 | 0.002153 |
| TMCC3 | 0.938134 | 6.148349 | 4.341486 | 2.75E-05 | 0.002186 |
| MUC5AC | 1.31596 | 12.66856 | 4.336572 | 2.80E-05 | 0.002222 |
| CATSPERB | 0.657989 | 6.566781 | 4.322807 | 2.96E-05 | 0.002323 |
| SERPINB5 | 0.81967 | 7.953503 | 4.301992 | 3.21E-05 | 0.002498 |
| ATP6V0A4 | 0.796509 | 8.858919 | 4.28965 | 3.38E-05 | 0.002609 |
| CREB3L1 | 0.648934 | 9.57515 | 4.273 | 3.61E-05 | 0.002757 |
| KLHL29 | -0.64635 | 5.848428 | -4.2613 | 3.78E-05 | 0.00285 |
| PHYHIPL | 0.634988 | 6.147015 | 4.258531 | 3.82E-05 | 0.002872 |
| DSC3 | 0.807698 | 6.573615 | 4.254393 | 3.88E-05 | 0.002907 |
| MFSD2A | 0.585304 | 7.791415 | 4.253368 | 3.90E-05 | 0.002907 |
| LINC01187 | 0.685844 | 6.000101 | 4.242925 | 4.06E-05 | 0.002996 |
| LRRC31 | 1.124171 | 7.609003 | 4.241171 | 4.09E-05 | 0.002999 |
| LOC100507560 | -1.23877 | 7.057401 | -4.23171 | 4.24E-05 | 0.003063 |
| ALDH3A1 | 1.388291 | 13.58564 | 4.223392 | 4.38E-05 | 0.003135 |
| EGF | 1.213918 | 6.226054 | 4.21351 | 4.56E-05 | 0.00322 |
| TXNRD1 | 0.630478 | 11.71742 | 4.209817 | 4.62E-05 | 0.003257 |
| C10orf10 | -0.85794 | 6.102873 | -4.20892 | 4.64E-05 | 0.003258 |
| TUB | -0.61255 | 6.726655 | -4.20691 | 4.67E-05 | 0.003274 |
| LOC100505918 | -0.65399 | 6.783165 | -4.20597 | 4.69E-05 | 0.003276 |
| FMO2 | -0.61831 | 10.92422 | -4.19471 | 4.90E-05 | 0.003373 |
| TMEM117 | 1.161229 | 6.192746 | 4.189659 | 5.00E-05 | 0.003392 |
| PPP1R16B | -1.19035 | 7.344165 | -4.18803 | 5.03E-05 | 0.003402 |
| CES1P1 | -1.41043 | 7.124577 | -4.18225 | 5.15E-05 | 0.003444 |
| PCDHB10 | 1.09067 | 4.819126 | 4.179204 | 5.21E-05 | 0.003461 |
| PHEX | 1.016714 | 7.725932 | 4.160077 | 5.61E-05 | 0.003678 |
| SLC7A5 | 0.819558 | 6.555333 | 4.159884 | 5.61E-05 | 0.003678 |
| CTCFL | 1.058866 | 4.066799 | 4.1582 | 5.65E-05 | 0.003692 |
| LOC338667 | -1.01461 | 6.490027 | -4.15477 | 5.73E-05 | 0.003731 |
| FAM65C | -0.85326 | 5.614009 | -4.14986 | 5.84E-05 | 0.003782 |
| CTB-167B5.2 | -0.75633 | 7.819473 | -4.13612 | 6.16E-05 | 0.003944 |
| APOLD1 | 0.619108 | 6.733546 | 4.131495 | 6.27E-05 | 0.003994 |
| PLD4 | 0.630449 | 5.405808 | 4.110737 | 6.79E-05 | 0.004257 |
| ANHX | -1.10532 | 5.68201 | -4.10803 | 6.86E-05 | 0.004278 |
| PRKCA | -0.7271 | 5.383786 | -4.10648 | 6.90E-05 | 0.004284 |
| AKR1C3 | 0.851729 | 12.82835 | 4.10434 | 6.96E-05 | 0.004305 |
| SAA1 | -1.57866 | 8.958943 | -4.10149 | 7.04E-05 | 0.004329 |
| THSD7A | -0.84433 | 6.075375 | -4.09885 | 7.11E-05 | 0.004362 |
| SLC16A4 | 0.745124 | 6.142723 | 4.081922 | 7.58E-05 | 0.004594 |
| PANK1 | -0.59571 | 8.073122 | -4.07925 | 7.66E-05 | 0.004629 |
| POU5F1 | -1.03964 | 6.889456 | -4.07199 | 7.88E-05 | 0.004687 |
| SHISA9 | -0.80704 | 5.734662 | -4.06987 | 7.94E-05 | 0.004713 |
| AKR1C2 | 0.951878 | 13.31135 | 4.065264 | 8.08E-05 | 0.004764 |
| CD44 | 0.588124 | 8.345056 | 4.065093 | 8.09E-05 | 0.004764 |
| PXDN | 0.609833 | 6.504441 | 4.055773 | 8.38E-05 | 0.00485 |
| BTBD11 | 0.619036 | 6.015547 | 4.043318 | 8.79E-05 | 0.005032 |
| CYP3A5 | 0.686122 | 6.215716 | 4.04288 | 8.80E-05 | 0.005032 |
| SCGB3A2 | -2.74216 | 8.901721 | -4.03894 | 8.94E-05 | 0.005083 |
| LOC101928620 | -0.62276 | 10.76266 | -4.01979 | 9.61E-05 | 0.005362 |
| LINC00869 | -0.71325 | 7.123143 | -4.01811 | 9.67E-05 | 0.005371 |
| LOC284630 | -0.94179 | 6.430653 | -4.01806 | 9.67E-05 | 0.005371 |
| CPNE4 | 0.94962 | 5.079742 | 4.013312 | 9.85E-05 | 0.005426 |
| C22orf42 | 0.852805 | 5.770721 | 4.012726 | 9.87E-05 | 0.005426 |
| FABP6 | -0.63908 | 7.732647 | -4.01171 | 9.91E-05 | 0.005426 |
| RIMS1 | -0.60219 | 7.178079 | -4.01151 | 9.92E-05 | 0.005426 |
| PLN | 0.815285 | 3.299252 | 4.010223 | 9.96E-05 | 0.005426 |
| BCL2A1 | 1.178745 | 8.900822 | 4.009394 | 1.00E-04 | 0.005426 |
| RPL13AP17 | -0.85099 | 7.813971 | -4.00916 | 0.0001 | 0.005426 |
| LSS | -0.73555 | 7.111867 | -4.00442 | 0.000102 | 0.005471 |
| TIMP4 | 0.970404 | 8.951056 | 3.992237 | 0.000107 | 0.005653 |
| DPP10 | 0.834646 | 6.278494 | 3.983635 | 0.00011 | 0.0058 |
| TMED2 | -0.60135 | 11.31611 | -3.98093 | 0.000111 | 0.00582 |
| CYP2F1 | -0.66378 | 11.28717 | -3.98032 | 0.000112 | 0.00582 |
| BAALC | 0.680786 | 7.616461 | 3.980081 | 0.000112 | 0.00582 |
| NXPH3 | -0.73994 | 6.159979 | -3.97762 | 0.000113 | 0.005841 |
| TSPAN5 | 0.616304 | 7.089487 | 3.971186 | 0.000115 | 0.00597 |
| SPINK5 | 0.767056 | 8.143098 | 3.969452 | 0.000116 | 0.005996 |
| CYP4F3 | 1.025419 | 7.995149 | 3.961495 | 0.00012 | 0.006124 |
| ATF6B | -0.60102 | 6.555602 | -3.95704 | 0.000122 | 0.006173 |
| CNN3 | -0.72353 | 10.12995 | -3.95392 | 0.000123 | 0.006209 |
| ZNF680 | -0.61226 | 8.737051 | -3.9535 | 0.000123 | 0.006209 |
| LOC344887 | 1.274083 | 6.773277 | 3.953189 | 0.000124 | 0.006209 |
| IGSF6 | 0.963734 | 6.535179 | 3.94824 | 0.000126 | 0.006298 |
| OR13C4 | -0.79667 | 5.699779 | -3.94465 | 0.000128 | 0.006362 |
| LINC00928 | -0.8082 | 5.184024 | -3.94393 | 0.000128 | 0.006362 |
| BPIFA2 | 0.984763 | 5.715564 | 3.943437 | 0.000128 | 0.006362 |
| NR0B1 | 0.902695 | 5.606244 | 3.920965 | 0.000139 | 0.006808 |
| ME1 | 1.050549 | 9.207361 | 3.916382 | 0.000142 | 0.006875 |
| DIO1 | 0.587594 | 9.543556 | 3.911085 | 0.000145 | 0.006965 |
| FJX1 | -0.82253 | 7.064809 | -3.90686 | 0.000147 | 0.007003 |
| BRSK2 | -0.73279 | 4.492213 | -3.89392 | 0.000154 | 0.007229 |
| KRT40 | 0.865463 | 5.022702 | 3.88981 | 0.000156 | 0.007317 |
| SEMA5B | -1.12048 | 5.985781 | -3.8896 | 0.000157 | 0.007317 |
| CREBBP | -0.63296 | 6.93115 | -3.88201 | 0.000161 | 0.007495 |
| CYP27B1 | 0.803792 | 5.693176 | 3.880887 | 0.000162 | 0.007511 |
| OXR1 | -0.61932 | 9.370172 | -3.87969 | 0.000162 | 0.007515 |
| DEGS2 | -0.63626 | 10.13779 | -3.87818 | 0.000163 | 0.00754 |
| BC039487 | 1.149545 | 4.11454 | 3.877387 | 0.000164 | 0.00754 |
| SMIM1 | -1.11769 | 5.757969 | -3.87248 | 0.000167 | 0.007612 |
| LOC340184 | 0.832737 | 6.410692 | 3.852725 | 0.000179 | 0.008015 |
| GPRC5D | -0.89174 | 5.253394 | -3.84932 | 0.000182 | 0.008085 |
| EREG | 0.686096 | 4.752878 | 3.826857 | 0.000197 | 0.008631 |
| RP13-238F13.5 | 1.111789 | 4.710078 | 3.822655 | 0.0002 | 0.008749 |
| GPR87 | 0.644471 | 7.750717 | 3.812387 | 0.000208 | 0.008944 |
| HSPB8 | 0.646199 | 6.237745 | 3.811274 | 0.000209 | 0.008944 |
| HSD17B2 | -1.25392 | 6.521658 | -3.80302 | 0.000215 | 0.009126 |
| APOH | -0.72589 | 4.972143 | -3.79345 | 0.000223 | 0.009365 |
| ZNF672 | -0.63298 | 6.40025 | -3.79215 | 0.000224 | 0.009392 |
| ABHD2 | 0.600639 | 10.68559 | 3.790565 | 0.000225 | 0.009413 |
| NETO2 | 0.644106 | 5.736465 | 3.789729 | 0.000226 | 0.009424 |
| HEYL | -0.59557 | 6.162608 | -3.76097 | 0.000251 | 0.0101 |
| D2HGDH | -0.64254 | 8.617869 | -3.76049 | 0.000251 | 0.0101 |
| THBS1 | 0.696857 | 5.043696 | 3.758279 | 0.000253 | 0.010128 |
| DTNA | 0.598459 | 6.131827 | 3.753358 | 0.000258 | 0.010259 |
| PANX3 | 1.077308 | 3.934127 | 3.735322 | 0.000275 | 0.010818 |
| APOBEC3B | 0.713407 | 6.878289 | 3.734162 | 0.000276 | 0.010845 |
| ARNTL2 | 0.839929 | 6.212023 | 3.719996 | 0.000291 | 0.011279 |
| ATP6V1B1 | -0.58558 | 6.608965 | -3.71237 | 0.000299 | 0.011477 |
| QTRTD1 | 0.939377 | 4.940188 | 3.711764 | 0.000299 | 0.011478 |
| G6PD | 0.70253 | 8.441717 | 3.705777 | 0.000306 | 0.011693 |
| LINC01410 | 0.664891 | 6.850089 | 3.704916 | 0.000307 | 0.01171 |
| PP14571 | 0.64257 | 7.735024 | 3.70097 | 0.000311 | 0.011819 |
| EMR2 | 0.622374 | 6.422123 | 3.698209 | 0.000314 | 0.011878 |
| PNMA2 | -1.03639 | 6.512529 | -3.68771 | 0.000326 | 0.012292 |
| PTPRD | 0.670062 | 5.130774 | 3.686236 | 0.000328 | 0.012317 |
| CBR1 | 0.894504 | 10.70945 | 3.684259 | 0.00033 | 0.012365 |
| DPP4 | -0.71966 | 7.917971 | -3.68011 | 0.000335 | 0.012529 |
| FSCN1 | 0.652598 | 5.526067 | 3.670675 | 0.000347 | 0.012915 |
| CX3CL1 | -0.74477 | 8.710314 | -3.66957 | 0.000348 | 0.012936 |
| TEF | -0.71457 | 5.911567 | -3.66119 | 0.000359 | 0.013231 |
| DUSP5P1 | 1.07127 | 5.703309 | 3.654028 | 0.000368 | 0.013445 |
| ADH7 | 1.216261 | 11.5593 | 3.65322 | 0.000369 | 0.013445 |
| ATP13A4 | -0.60754 | 7.013798 | -3.65322 | 0.000369 | 0.013445 |
| SPDEF | 0.620743 | 9.449477 | 3.653152 | 0.000369 | 0.013445 |
| CD86 | 0.701794 | 6.841933 | 3.650117 | 0.000373 | 0.013526 |
| SAMSN1 | 0.825314 | 7.624541 | 3.632867 | 0.000396 | 0.014121 |
| SLC35G1 | 0.676797 | 5.459807 | 3.629366 | 0.000401 | 0.014191 |
| PNMA6A | -0.86281 | 6.276391 | -3.62811 | 0.000403 | 0.014191 |
| AC074212.6 | -0.6768 | 5.448886 | -3.62774 | 0.000404 | 0.014191 |
| TMC7 | 0.702765 | 4.989779 | 3.625262 | 0.000407 | 0.014275 |
| DNAJC12 | 0.924408 | 7.545416 | 3.623349 | 0.00041 | 0.014329 |
| BCAT1 | 0.852639 | 5.883093 | 3.619918 | 0.000415 | 0.014442 |
| TGFBI | 0.616678 | 9.806881 | 3.614121 | 0.000423 | 0.014683 |
| LAG3 | -0.83967 | 6.110064 | -3.61395 | 0.000424 | 0.014683 |
| RP11-203B7.1 | 0.937582 | 3.718983 | 3.610664 | 0.000429 | 0.014798 |
| CTHRC1 | 0.976868 | 4.933639 | 3.596893 | 0.00045 | 0.015379 |
| CGREF1 | 0.940333 | 4.658434 | 3.596105 | 0.000451 | 0.015381 |
| SLC6A13 | -0.61751 | 8.108814 | -3.59027 | 0.00046 | 0.015653 |
| RP11-524D16__A.3 | 0.900666 | 5.697437 | 3.589216 | 0.000462 | 0.015665 |
| ASB2 | -0.7257 | 6.195755 | -3.58087 | 0.000476 | 0.015879 |
| MEGF6 | -0.69508 | 6.055779 | -3.58081 | 0.000476 | 0.015879 |
| ERP27 | -0.76305 | 8.666222 | -3.57892 | 0.000479 | 0.015926 |
| TMEM200B | 0.65416 | 6.493498 | 3.57751 | 0.000481 | 0.015926 |
| SPIN2A | 0.797105 | 4.33778 | 3.576157 | 0.000484 | 0.015944 |
| LOC102724094 | 0.960091 | 7.872143 | 3.572845 | 0.000489 | 0.016005 |
| PCDH8 | 1.024886 | 3.497603 | 3.571611 | 0.000491 | 0.016005 |
| RCVRN | -0.80417 | 4.635638 | -3.56725 | 0.000499 | 0.016185 |
| ANKRD36C | 0.708242 | 6.570491 | 3.564344 | 0.000504 | 0.016237 |
| C1QTNF4 | -1.05201 | 4.512531 | -3.56144 | 0.000509 | 0.016326 |
| WTIP | -0.84584 | 6.586192 | -3.55851 | 0.000514 | 0.016326 |
| TFF1 | 0.991619 | 9.649758 | 3.556507 | 0.000518 | 0.016397 |
| DLX2 | -0.60847 | 6.050189 | -3.55602 | 0.000519 | 0.016397 |
| RP3-406C18.2 | -0.81923 | 4.675856 | -3.55371 | 0.000523 | 0.016507 |
| PROK2 | 1.358584 | 6.06209 | 3.551049 | 0.000528 | 0.016593 |
| CENPI | 0.589542 | 4.602727 | 3.54553 | 0.000538 | 0.016801 |
| FAM186A | 0.726189 | 4.740886 | 3.544575 | 0.00054 | 0.016834 |
| OSM | 0.794443 | 5.412043 | 3.536842 | 0.000555 | 0.017141 |
| SAMD1 | -0.69221 | 7.020218 | -3.5366 | 0.000555 | 0.017141 |
| RP11-96H17.1 | 0.948152 | 2.851192 | 3.536297 | 0.000556 | 0.017141 |
| TMEM221 | -0.7125 | 5.284157 | -3.53131 | 0.000565 | 0.017325 |
| FCN1 | 0.985939 | 6.805361 | 3.530496 | 0.000567 | 0.017351 |
| ANKRD20A1 | 0.668852 | 8.940695 | 3.528982 | 0.00057 | 0.017396 |
| SMIM17 | 1.080985 | 4.379584 | 3.526042 | 0.000576 | 0.017454 |
| VEPH1 | -0.88842 | 5.805446 | -3.51998 | 0.000588 | 0.017623 |
| FAM150B | 0.955009 | 3.774925 | 3.513188 | 0.000602 | 0.017968 |
| MMP7 | -0.88629 | 8.340684 | -3.51125 | 0.000606 | 0.018023 |
| MBNL2 | -0.59884 | 8.75198 | -3.51003 | 0.000608 | 0.018068 |
| DNASE1L2 | -0.67138 | 6.152642 | -3.50571 | 0.000618 | 0.018185 |
| HDX | 0.938346 | 4.661877 | 3.504104 | 0.000621 | 0.018239 |
| GPR183 | 0.697676 | 7.163376 | 3.502007 | 0.000625 | 0.018325 |
| IGKV1-37 | -0.85759 | 4.872832 | -3.4996 | 0.000631 | 0.018431 |
| CST6 | 0.595302 | 9.770752 | 3.495418 | 0.00064 | 0.0186 |
| SECTM1 | 0.61625 | 8.120718 | 3.495126 | 0.00064 | 0.0186 |
| BEND7 | 0.733761 | 4.874229 | 3.488706 | 0.000655 | 0.018943 |
| C15orf48 | 0.857074 | 9.538369 | 3.485661 | 0.000662 | 0.019048 |
| RP5-1119A7.11 | -0.70352 | 5.541921 | -3.47727 | 0.000681 | 0.019436 |
| LCAT | -0.70508 | 4.892957 | -3.47429 | 0.000688 | 0.019587 |
| LINGO2 | 0.635386 | 6.321378 | 3.471891 | 0.000694 | 0.0197 |
| DEPDC1 | 0.597065 | 5.225123 | 3.469562 | 0.000699 | 0.019785 |
| NPM1P22 | -0.66471 | 8.012422 | -3.46643 | 0.000707 | 0.01991 |
| FAM110B | -0.67693 | 5.832841 | -3.45828 | 0.000726 | 0.020243 |
| PRH1-PRR4 | 0.904777 | 8.773444 | 3.455619 | 0.000733 | 0.020378 |
| MKL2 | -0.60829 | 5.439737 | -3.45347 | 0.000738 | 0.020463 |
| LINC00942 | 0.891802 | 5.380843 | 3.45053 | 0.000746 | 0.020551 |
| EXOG | -0.58623 | 6.211802 | -3.44824 | 0.000752 | 0.020651 |
| OR10J1 | -0.74825 | 5.304452 | -3.44583 | 0.000758 | 0.020771 |
| EFCAB5 | 0.682519 | 4.496794 | 3.444628 | 0.000761 | 0.020808 |
| RP11-385F5.4 | -0.83511 | 5.647511 | -3.44362 | 0.000764 | 0.02083 |
| ITGB2-AS1 | -0.65046 | 6.441788 | -3.4372 | 0.00078 | 0.021215 |
| TRA@ | -0.72756 | 4.487476 | -3.43378 | 0.00079 | 0.021364 |
| DKFZp434L192 | 0.746236 | 3.037288 | 3.431843 | 0.000795 | 0.021454 |
| PHACTR3 | 0.871639 | 5.219159 | 3.429707 | 0.000801 | 0.02156 |
| FZD9 | -0.856 | 4.48472 | -3.42777 | 0.000806 | 0.021603 |
| HRH4 | 0.699001 | 4.555612 | 3.42594 | 0.000811 | 0.021635 |
| KIAA1211 | 0.707355 | 6.775073 | 3.4231 | 0.000819 | 0.021747 |
| CCRL2 | 0.963463 | 6.594313 | 3.421604 | 0.000823 | 0.021807 |
| CHIA | -0.83457 | 5.260596 | -3.41972 | 0.000828 | 0.021821 |
| FPR3 | 0.689419 | 7.281708 | 3.41965 | 0.000828 | 0.021821 |
| JAKMIP3 | 1.149753 | 6.184754 | 3.419298 | 0.000829 | 0.021821 |
| SPRR1A | 0.594724 | 6.166703 | 3.410633 | 0.000854 | 0.022302 |
| LINC00862 | 0.910973 | 4.206741 | 3.408831 | 0.000859 | 0.022363 |
| SFRP2 | 1.10328 | 6.481291 | 3.400689 | 0.000883 | 0.022883 |
| HOTS | 1.320111 | 5.646461 | 3.3895 | 0.000917 | 0.023629 |
| MIR302B | 0.725482 | 7.288324 | 3.383529 | 0.000935 | 0.023949 |
| TANC2 | -0.69093 | 5.966326 | -3.38289 | 0.000938 | 0.023969 |
| TSHB | -0.79695 | 5.225077 | -3.3817 | 0.000941 | 0.024017 |
| TMOD1 | -0.65468 | 5.942003 | -3.38056 | 0.000945 | 0.024065 |
| LINC00277 | -0.89115 | 5.042057 | -3.38012 | 0.000946 | 0.024065 |
| CD163L1 | 0.730907 | 5.555117 | 3.379782 | 0.000947 | 0.024067 |
| HSD17B6 | 0.758048 | 6.074504 | 3.378572 | 0.000951 | 0.024112 |
| KIR2DL3 | -0.77975 | 5.157796 | -3.37265 | 0.00097 | 0.024464 |
| ZFP36L2 | -0.62476 | 8.316525 | -3.37213 | 0.000972 | 0.024464 |
| LRIG1 | -0.60611 | 7.215337 | -3.36761 | 0.000987 | 0.024718 |
| HOXB-AS1 | 0.642623 | 7.257625 | 3.351442 | 0.001041 | 0.025894 |
| LOC101929064 | -0.77945 | 5.231512 | -3.35025 | 0.001046 | 0.025957 |
| CTXN3 | -0.64348 | 4.546831 | -3.34759 | 0.001055 | 0.026118 |
| BHMT2 | 0.664416 | 4.708317 | 3.345811 | 0.001061 | 0.026215 |
| NCAPG | 0.605176 | 5.509963 | 3.344642 | 0.001065 | 0.026264 |
| CYTIP | 0.604477 | 8.104735 | 3.338482 | 0.001087 | 0.026638 |
| FOXI1 | 0.776717 | 5.995204 | 3.337749 | 0.00109 | 0.026675 |
| DAB2 | 0.720411 | 7.766481 | 3.326296 | 0.001132 | 0.027495 |
| CD163 | 0.884989 | 8.817547 | 3.325909 | 0.001134 | 0.027495 |
| MAGEB3 | 0.826837 | 4.130274 | 3.32548 | 0.001135 | 0.027495 |
| AKAP12 | 0.588091 | 5.10264 | 3.323641 | 0.001142 | 0.02762 |
| SIX3 | 0.826111 | 5.588338 | 3.323106 | 0.001144 | 0.02762 |
| HLA-DOA | -0.59905 | 6.173529 | -3.32105 | 0.001152 | 0.027697 |
| FLJ11710 | -0.69149 | 6.581853 | -3.32092 | 0.001153 | 0.027697 |
| IL20RB | 0.617873 | 6.366086 | 3.320295 | 0.001155 | 0.027697 |
| AKR1C1 | 1.021629 | 10.81526 | 3.320088 | 0.001156 | 0.027697 |
| RP11-295M18.6 | 0.849984 | 5.188429 | 3.319291 | 0.001159 | 0.027719 |
| ZNF815P | -0.75607 | 4.683899 | -3.31738 | 0.001166 | 0.027814 |
| TMEM72-AS1 | -0.80992 | 5.64551 | -3.30854 | 0.001201 | 0.028376 |
| FOLH1B | 0.702403 | 6.85094 | 3.30806 | 0.001203 | 0.028389 |
| KCNT1 | -0.62695 | 4.968487 | -3.30542 | 0.001213 | 0.028609 |
| ZNRF4 | -0.75275 | 5.520942 | -3.30411 | 0.001218 | 0.028688 |
| KIF12 | -0.84676 | 5.41551 | -3.30187 | 0.001227 | 0.028829 |
| LOC100147773 | -0.66513 | 6.288423 | -3.29971 | 0.001236 | 0.028977 |
| FGF12 | 0.725907 | 5.669664 | 3.296309 | 0.00125 | 0.029215 |
| IL33 | -0.63402 | 9.901372 | -3.29336 | 0.001262 | 0.029421 |
| LOC340357 | -0.78948 | 4.046554 | -3.28855 | 0.001283 | 0.029851 |
| LOC339539 | -0.80976 | 5.198279 | -3.28816 | 0.001284 | 0.02986 |
| JAM3 | -0.65139 | 5.391343 | -3.28656 | 0.001291 | 0.029957 |
| RP11-687F6.1 | 0.896261 | 4.643637 | 3.285737 | 0.001294 | 0.030009 |
| RP13-20L14.1 | -0.83372 | 4.818625 | -3.27788 | 0.001328 | 0.03055 |
| CCDC14 | 0.642915 | 4.500907 | 3.276133 | 0.001336 | 0.030639 |
| CYP4F2 | 0.891085 | 5.337163 | 3.274339 | 0.001344 | 0.030654 |
| LOC101927690 | 0.922919 | 4.618326 | 3.273888 | 0.001346 | 0.030654 |
| LOC101929180 | -0.73973 | 5.418799 | -3.27289 | 0.00135 | 0.030691 |
| LRRC75A | -0.7025 | 4.12373 | -3.27235 | 0.001353 | 0.030696 |
| EXO1 | -0.79382 | 5.393395 | -3.26823 | 0.001371 | 0.030952 |
| CYSLTR1 | 0.611934 | 6.114791 | 3.267803 | 0.001373 | 0.030965 |
| DKK1 | 0.739163 | 6.149352 | 3.266384 | 0.001379 | 0.03104 |
| LOC283335 | -0.96975 | 5.897505 | -3.2625 | 0.001397 | 0.031174 |
| PIR | 0.660086 | 11.31572 | 3.261616 | 0.001401 | 0.031205 |
| SLC34A2 | -0.58565 | 11.49024 | -3.26028 | 0.001407 | 0.031253 |
| H19 | 1.178538 | 9.150045 | 3.259305 | 0.001412 | 0.031322 |
| PRKG1 | 0.732016 | 4.971657 | 3.25882 | 0.001414 | 0.031342 |
| SCD | -0.63774 | 7.348421 | -3.25381 | 0.001437 | 0.031619 |
| SRGN | 0.823055 | 10.63296 | 3.251144 | 0.00145 | 0.031835 |
| GIPC3 | -0.66563 | 5.629106 | -3.24943 | 0.001458 | 0.031925 |
| GLS2 | -0.60069 | 5.234673 | -3.24157 | 0.001496 | 0.032507 |
| SLC26A9 | 0.593794 | 6.411573 | 3.240092 | 0.001503 | 0.032632 |
| CCR1 | 0.685784 | 7.701222 | 3.236521 | 0.00152 | 0.032825 |
| LOC101928386 | 0.793105 | 2.734621 | 3.235992 | 0.001523 | 0.032825 |
| AZGP1 | 0.755368 | 8.206902 | 3.232164 | 0.001542 | 0.033114 |
| MC2R | 0.631868 | 4.259924 | 3.231595 | 0.001545 | 0.033145 |
| CTA-246H3.12 | -0.6638 | 4.557333 | -3.23013 | 0.001552 | 0.033272 |
| CEACAM19 | -0.88176 | 6.085358 | -3.22347 | 0.001586 | 0.033813 |
| PLK2 | -0.61607 | 8.200299 | -3.22058 | 0.001601 | 0.034099 |
| TNIP3 | 1.002943 | 4.087285 | 3.210192 | 0.001656 | 0.034739 |
| CBR3 | 0.650189 | 9.222966 | 3.208716 | 0.001664 | 0.03477 |
| VGLL3 | -0.97008 | 6.803554 | -3.20868 | 0.001664 | 0.03477 |
| BC040412 | -0.64139 | 6.646564 | -3.20703 | 0.001673 | 0.034925 |
| LY6D | 1.298393 | 6.861791 | 3.205831 | 0.001679 | 0.035029 |
| FRRS1 | 0.854178 | 4.219119 | 3.205297 | 0.001682 | 0.035036 |
| TOX | -0.59882 | 7.182016 | -3.20508 | 0.001683 | 0.035036 |
| SGCA | -0.74909 | 3.878796 | -3.19348 | 0.001747 | 0.035912 |
| AGPAT9 | 0.682442 | 9.04606 | 3.189679 | 0.001769 | 0.036054 |
| GPRIN1 | -0.63393 | 4.59982 | -3.18962 | 0.001769 | 0.036054 |
| NR4A3 | 0.685434 | 7.326069 | 3.187584 | 0.001781 | 0.036128 |
| KCNH8 | 0.889286 | 4.617738 | 3.186977 | 0.001784 | 0.036128 |
| GPR34 | 0.641817 | 7.421165 | 3.186082 | 0.001789 | 0.036156 |
| ERVMER61-1 | 0.804379 | 2.939112 | 3.18499 | 0.001796 | 0.036221 |
| TAS2R1 | 0.74886 | 5.405027 | 3.184781 | 0.001797 | 0.036221 |
| LSAMP | -0.58775 | 4.081119 | -3.18272 | 0.001809 | 0.036369 |
| CYP26B1 | -0.67475 | 4.131344 | -3.17999 | 0.001825 | 0.03662 |
| LOC102724483 | -0.68051 | 6.145277 | -3.17877 | 0.001832 | 0.036706 |
| LINC00630 | 0.68758 | 4.837952 | 3.171977 | 0.001872 | 0.037226 |
| TFF3 | 0.624656 | 13.4466 | 3.166638 | 0.001905 | 0.037637 |
| CACNA2D3 | 0.745989 | 5.939327 | 3.165364 | 0.001912 | 0.037637 |
| DDX17 | -0.75756 | 10.60173 | -3.1645 | 0.001918 | 0.03771 |
| ABCA12 | 0.844947 | 4.959829 | 3.1632 | 0.001926 | 0.037835 |
| SPATA1 | 0.653354 | 5.608133 | 3.158771 | 0.001953 | 0.038181 |
| GAPDHS | 0.609611 | 4.081902 | 3.157644 | 0.00196 | 0.038255 |
| WIF1 | -0.87683 | 9.211331 | -3.15725 | 0.001963 | 0.038271 |
| PRAC1 | 0.905927 | 4.581915 | 3.154268 | 0.001981 | 0.038508 |
| APOBEC3A | 0.993914 | 6.250527 | 3.15163 | 0.001998 | 0.038736 |
| MFI2-AS1 | -0.6327 | 6.091785 | -3.14837 | 0.002019 | 0.039011 |
| RP11-1007O24.3 | -0.87105 | 5.196432 | -3.14112 | 0.002066 | 0.039561 |
| RP11-44N11.3 | 0.773485 | 5.120494 | 3.135524 | 0.002103 | 0.040172 |
| MILR1 | 0.793463 | 4.686947 | 3.133956 | 0.002114 | 0.040296 |
| LOC100506446 | -0.63502 | 5.915896 | -3.1185 | 0.00222 | 0.041516 |
| RTKN2 | 0.800889 | 4.431607 | 3.11466 | 0.002247 | 0.041822 |
| ASPM | 0.614867 | 4.99772 | 3.114226 | 0.00225 | 0.041846 |
| LINC00595 | 0.683122 | 6.659202 | 3.11326 | 0.002257 | 0.041889 |
| LOC101927363 | 0.720796 | 3.404252 | 3.113255 | 0.002257 | 0.041889 |
| ST7-AS1 | -0.67826 | 5.594803 | -3.11245 | 0.002263 | 0.041948 |
| IFI30 | 0.742425 | 11.64835 | 3.111275 | 0.002271 | 0.04207 |
| RP11-38P22.2 | 0.713104 | 5.824232 | 3.110456 | 0.002277 | 0.042146 |
| L2HGDH | -0.60265 | 6.418953 | -3.10786 | 0.002296 | 0.04233 |
| MIR3185 | -0.66157 | 6.19982 | -3.10782 | 0.002296 | 0.04233 |
| ORM1 | 0.657982 | 4.192394 | 3.097338 | 0.002373 | 0.043307 |
| PDE4B | 0.666938 | 5.963827 | 3.096109 | 0.002382 | 0.043407 |
| GPR112 | 0.674163 | 3.947551 | 3.094872 | 0.002392 | 0.043497 |
| OSTM1-AS1 | 0.712185 | 3.280541 | 3.094454 | 0.002395 | 0.043497 |
| RP11-524C21.2 | 0.702984 | 5.870994 | 3.093315 | 0.002403 | 0.043586 |
| RP11-809F4.3 | 0.917228 | 3.474879 | 3.092314 | 0.002411 | 0.043689 |
| COCH | 0.648576 | 5.210685 | 3.091234 | 0.002419 | 0.04377 |
| BC024027 | -0.75646 | 4.451862 | -3.08844 | 0.00244 | 0.043888 |
| LOC100506538 | -0.68086 | 6.061908 | -3.08113 | 0.002497 | 0.044655 |
| CYP4F11 | 0.924429 | 7.96224 | 3.080927 | 0.002499 | 0.044655 |
| LOC101928134 | -0.66787 | 5.581995 | -3.07858 | 0.002517 | 0.044878 |
| TFPI2 | -0.77605 | 6.278037 | -3.07843 | 0.002518 | 0.044878 |
| LOC283194 | -0.65245 | 5.463719 | -3.07799 | 0.002522 | 0.044896 |
| NMU | 0.716603 | 6.302959 | 3.076543 | 0.002533 | 0.045032 |
| GYS2 | 0.944045 | 4.605627 | 3.0734 | 0.002558 | 0.045339 |
| DCTN1-AS1 | 0.712433 | 6.68167 | 3.072591 | 0.002565 | 0.04542 |
| AREL1 | -0.60893 | 5.624993 | -3.06749 | 0.002606 | 0.045908 |
| P2RY1 | 0.746999 | 5.123789 | 3.065186 | 0.002625 | 0.046168 |
| REG3G | 0.763408 | 4.987443 | 3.064921 | 0.002627 | 0.046171 |
| INPP5B | -0.74697 | 6.522253 | -3.05763 | 0.002688 | 0.046913 |
| NCF1 | 0.764413 | 6.84459 | 3.056342 | 0.002698 | 0.046965 |
| PBK | 0.74096 | 5.647649 | 3.055277 | 0.002707 | 0.047051 |
| TMEM95 | -0.74365 | 6.45312 | -3.05032 | 0.00275 | 0.047535 |
| IGKC | -0.60091 | 5.604172 | -3.0453 | 0.002793 | 0.04807 |
| NPY2R | -0.59851 | 4.766053 | -3.04367 | 0.002807 | 0.048188 |
| LRP2 | -0.62499 | 6.814243 | -3.0299 | 0.00293 | 0.049474 |
| TRIM6 | 0.702967 | 6.355751 | 3.026196 | 0.002963 | 0.049864 |
