# Supplementary material for: Analysis of immune characteristics and inflammatory mechanisms in COPD patients: a multi-layered study combining bulk and single-cell transcriptome analysis and machine learning
Source: Front Med (Lausanne). 2025 Jul 21;12:1592802. doi: 10.3389/fmed.2025.1592802 (PMC12318759; doi:10.3389/fmed.2025.1592802)
Supplement: Supplementary file 2 [file Table_2.docx]

**Supplementary Table 2. GO Enrichment Analysis Results of Differentially Expressed Genes.**

| **ONTOLOGY** | **ID** | **Description** | **GeneRatio** | **pvalue** | **p.adjust** | **qvalue** | **Count** |
| --- | --- | --- | --- | --- | --- | --- | --- |
| BP | GO:0042060 | wound healing | 28/342 | 1.21E-08 | 4.62E-05 | 3.97E-05 | 28 |
| BP | GO:0071466 | cellular response to xenobiotic stimulus | 16/342 | 4.42E-07 | 0.000468 | 0.000402 | 16 |
| BP | GO:0009913 | epidermal cell differentiation | 18/342 | 5.14E-07 | 0.000468 | 0.000402 | 18 |
| BP | GO:0042445 | hormone metabolic process | 18/342 | 5.46E-07 | 0.000468 | 0.000402 | 18 |
| BP | GO:0019369 | arachidonic acid metabolic process | 9/342 | 7.61E-07 | 0.000468 | 0.000402 | 9 |
| BP | GO:0030216 | keratinocyte differentiation | 15/342 | 8.06E-07 | 0.000468 | 0.000402 | 15 |
| BP | GO:0034350 | regulation of glial cell apoptotic process | 5/342 | 8.58E-07 | 0.000468 | 0.000402 | 5 |
| BP | GO:0007584 | response to nutrient | 14/342 | 1.16E-06 | 0.000517 | 0.000444 | 14 |
| BP | GO:0033559 | unsaturated fatty acid metabolic process | 12/342 | 1.26E-06 | 0.000517 | 0.000444 | 12 |
| BP | GO:0120254 | olefinic compound metabolic process | 14/342 | 1.35E-06 | 0.000517 | 0.000444 | 14 |
| BP | GO:0006766 | vitamin metabolic process | 11/342 | 3.67E-06 | 0.001273 | 0.001094 | 11 |
| BP | GO:0043588 | skin development | 19/342 | 4.62E-06 | 0.001471 | 0.001264 | 19 |
| BP | GO:0008544 | epidermis development | 21/342 | 6.61E-06 | 0.001942 | 0.001669 | 21 |
| BP | GO:0034349 | glial cell apoptotic process | 5/342 | 7.52E-06 | 0.002052 | 0.001763 | 5 |
| BP | GO:0010466 | negative regulation of peptidase activity | 16/342 | 8.10E-06 | 0.002064 | 0.001773 | 16 |
| BP | GO:0043648 | dicarboxylic acid metabolic process | 10/342 | 9.74E-06 | 0.002325 | 0.001998 | 10 |
| BP | GO:0006775 | fat-soluble vitamin metabolic process | 7/342 | 1.21E-05 | 0.002623 | 0.002254 | 7 |
| BP | GO:0045861 | negative regulation of proteolysis | 19/342 | 1.24E-05 | 0.002623 | 0.002254 | 19 |
| BP | GO:0006721 | terpenoid metabolic process | 10/342 | 1.41E-05 | 0.002829 | 0.002431 | 10 |
| BP | GO:0002237 | response to molecule of bacterial origin | 20/342 | 1.57E-05 | 0.002993 | 0.002571 | 20 |
| BP | GO:0006805 | xenobiotic metabolic process | 11/342 | 1.69E-05 | 0.003076 | 0.002643 | 11 |
| BP | GO:0006690 | icosanoid metabolic process | 11/342 | 1.97E-05 | 0.00342 | 0.002938 | 11 |
| BP | GO:0032496 | response to lipopolysaccharide | 19/342 | 2.29E-05 | 0.003811 | 0.003275 | 19 |
| BP | GO:0001523 | retinoid metabolic process | 9/342 | 2.59E-05 | 0.004125 | 0.003545 | 9 |
| BP | GO:0016101 | diterpenoid metabolic process | 9/342 | 3.43E-05 | 0.005036 | 0.004327 | 9 |
| BP | GO:0033273 | response to vitamin | 9/342 | 3.43E-05 | 0.005036 | 0.004327 | 9 |
| BP | GO:0050878 | regulation of body fluid levels | 19/342 | 4.26E-05 | 0.006026 | 0.005177 | 19 |
| BP | GO:0042572 | retinol metabolic process | 7/342 | 4.96E-05 | 0.006763 | 0.005811 | 7 |
| BP | GO:1903035 | negative regulation of response to wounding | 9/342 | 5.32E-05 | 0.007014 | 0.006026 | 9 |
| BP | GO:2000179 | positive regulation of neural precursor cell proliferation | 7/342 | 5.61E-05 | 0.007139 | 0.006134 | 7 |
| BP | GO:0006720 | isoprenoid metabolic process | 10/342 | 6.95E-05 | 0.008568 | 0.007361 | 10 |
| BP | GO:0007596 | blood coagulation | 14/342 | 7.23E-05 | 0.008624 | 0.007409 | 14 |
| BP | GO:1903034 | regulation of response to wounding | 12/342 | 7.45E-05 | 0.008624 | 0.007409 | 12 |
| BP | GO:0030282 | bone mineralization | 10/342 | 8.01E-05 | 0.008994 | 0.007728 | 10 |
| BP | GO:0032102 | negative regulation of response to external stimulus | 21/342 | 8.49E-05 | 0.009261 | 0.007957 | 21 |
| BP | GO:0031214 | biomineral tissue development | 12/342 | 8.81E-05 | 0.009351 | 0.008034 | 12 |
| BP | GO:0050817 | coagulation | 14/342 | 9.15E-05 | 0.009447 | 0.008116 | 14 |
| BP | GO:0007599 | hemostasis | 14/342 | 9.58E-05 | 0.009634 | 0.008278 | 14 |
| BP | GO:0008202 | steroid metabolic process | 17/342 | 0.000107 | 0.010414 | 0.008947 | 17 |
| BP | GO:0019748 | secondary metabolic process | 7/342 | 0.000111 | 0.010414 | 0.008947 | 7 |
| BP | GO:0001819 | positive regulation of cytokine production | 22/342 | 0.000112 | 0.010414 | 0.008947 | 22 |
| BP | GO:0006631 | fatty acid metabolic process | 19/342 | 0.000135 | 0.012299 | 0.010567 | 19 |
| BP | GO:0010951 | negative regulation of endopeptidase activity | 11/342 | 0.000186 | 0.016431 | 0.014117 | 11 |
| BP | GO:0042180 | cellular ketone metabolic process | 13/342 | 0.000189 | 0.016431 | 0.014117 | 13 |
| BP | GO:0001676 | long-chain fatty acid metabolic process | 9/342 | 0.000196 | 0.016555 | 0.014224 | 9 |
| BP | GO:0009410 | response to xenobiotic stimulus | 20/342 | 0.000199 | 0.016555 | 0.014224 | 20 |
| BP | GO:0009404 | toxin metabolic process | 4/342 | 0.000221 | 0.017318 | 0.014879 | 4 |
| BP | GO:0061041 | regulation of wound healing | 10/342 | 0.000224 | 0.017318 | 0.014879 | 10 |
| BP | GO:0006959 | humoral immune response | 14/342 | 0.000229 | 0.017318 | 0.014879 | 14 |
| BP | GO:2000177 | regulation of neural precursor cell proliferation | 8/342 | 0.000232 | 0.017318 | 0.014879 | 8 |
| BP | GO:0051346 | negative regulation of hydrolase activity | 17/342 | 0.000235 | 0.017318 | 0.014879 | 17 |
| BP | GO:0002673 | regulation of acute inflammatory response | 6/342 | 0.000236 | 0.017318 | 0.014879 | 6 |
| BP | GO:0001516 | prostaglandin biosynthetic process | 5/342 | 0.000272 | 0.019263 | 0.016551 | 5 |
| BP | GO:0046457 | prostanoid biosynthetic process | 5/342 | 0.000272 | 0.019263 | 0.016551 | 5 |
| BP | GO:0006692 | prostanoid metabolic process | 6/342 | 0.000296 | 0.020192 | 0.017349 | 6 |
| BP | GO:0006693 | prostaglandin metabolic process | 6/342 | 0.000296 | 0.020192 | 0.017349 | 6 |
| BP | GO:0042573 | retinoic acid metabolic process | 5/342 | 0.000316 | 0.021187 | 0.018204 | 5 |
| BP | GO:0006636 | unsaturated fatty acid biosynthetic process | 6/342 | 0.000368 | 0.02422 | 0.02081 | 6 |
| BP | GO:0050766 | positive regulation of phagocytosis | 7/342 | 0.000383 | 0.024795 | 0.021304 | 7 |
| BP | GO:0072593 | reactive oxygen species metabolic process | 13/342 | 0.000464 | 0.02952 | 0.025364 | 13 |
| BP | GO:0002688 | regulation of leukocyte chemotaxis | 9/342 | 0.000537 | 0.033239 | 0.028559 | 9 |
| BP | GO:0030595 | leukocyte chemotaxis | 13/342 | 0.000543 | 0.033239 | 0.028559 | 13 |
| BP | GO:0070167 | regulation of biomineral tissue development | 8/342 | 0.000549 | 0.033239 | 0.028559 | 8 |
| BP | GO:0031667 | response to nutrient levels | 20/342 | 0.000561 | 0.033239 | 0.028559 | 20 |
| BP | GO:0048545 | response to steroid hormone | 16/342 | 0.00057 | 0.033239 | 0.028559 | 16 |
| BP | GO:0061045 | negative regulation of wound healing | 7/342 | 0.000574 | 0.033239 | 0.028559 | 7 |
| BP | GO:1901661 | quinone metabolic process | 5/342 | 0.00062 | 0.035363 | 0.030384 | 5 |
| BP | GO:0030500 | regulation of bone mineralization | 7/342 | 0.000669 | 0.036581 | 0.03143 | 7 |
| BP | GO:0008300 | isoprenoid catabolic process | 3/342 | 0.00067 | 0.036581 | 0.03143 | 3 |
| BP | GO:0097267 | omega-hydroxylase P450 pathway | 3/342 | 0.00067 | 0.036581 | 0.03143 | 3 |
| BP | GO:0071621 | granulocyte chemotaxis | 9/342 | 0.000712 | 0.038333 | 0.032935 | 9 |
| BP | GO:0016485 | protein processing | 13/342 | 0.000764 | 0.040548 | 0.034839 | 13 |
| BP | GO:0061844 | antimicrobial humoral immune response mediated by antimicrobial peptide | 7/342 | 0.000776 | 0.040628 | 0.034908 | 7 |
| BP | GO:0045604 | regulation of epidermal cell differentiation | 6/342 | 0.0008 | 0.041006 | 0.035232 | 6 |
| BP | GO:0030593 | neutrophil chemotaxis | 8/342 | 0.000805 | 0.041006 | 0.035232 | 8 |
| BP | GO:0031424 | keratinization | 7/342 | 0.000835 | 0.04196 | 0.036051 | 7 |
| BP | GO:0051960 | regulation of nervous system development | 19/342 | 0.00094 | 0.046628 | 0.040062 | 19 |
| CC | GO:0001533 | cornified envelope | 8/369 | 1.45E-05 | 0.004701 | 0.004461 | 8 |
| CC | GO:0045177 | apical part of cell | 21/369 | 0.000128 | 0.013874 | 0.013166 | 21 |
| CC | GO:0016324 | apical plasma membrane | 19/369 | 0.000128 | 0.013874 | 0.013166 | 19 |
| CC | GO:0031091 | platelet alpha granule | 8/369 | 0.000328 | 0.026669 | 0.025308 | 8 |
| MF | GO:0016614 | oxidoreductase activity, acting on CH-OH group of donors | 16/354 | 5.99E-09 | 3.50E-06 | 3.06E-06 | 16 |
| MF | GO:0048018 | receptor ligand activity | 28/354 | 4.36E-07 | 0.000127 | 0.000111 | 28 |
| MF | GO:0046906 | tetrapyrrole binding | 14/354 | 9.94E-07 | 0.000194 | 0.000169 | 14 |
| MF | GO:0020037 | heme binding | 13/354 | 2.53E-06 | 0.00037 | 0.000323 | 13 |
| MF | GO:0004497 | monooxygenase activity | 11/354 | 4.78E-06 | 0.000559 | 0.000488 | 11 |
| MF | GO:0070330 | aromatase activity | 6/354 | 6.38E-06 | 0.000622 | 0.000543 | 6 |
| MF | GO:0016705 | oxidoreductase activity, acting on paired donors, with incorporation or reduction of molecular oxygen | 14/354 | 8.90E-06 | 0.000734 | 0.00064 | 14 |
| MF | GO:0016709 | oxidoreductase activity, acting on paired donors, with incorporation or reduction of molecular oxygen, NAD(P)H as one donor, and incorporation of one atom of oxygen | 7/354 | 1.00E-05 | 0.000734 | 0.00064 | 7 |
| MF | GO:0061135 | endopeptidase regulator activity | 14/354 | 1.66E-05 | 0.001081 | 0.000943 | 14 |
| MF | GO:0016616 | oxidoreductase activity, acting on the CH-OH group of donors, NAD or NADP as acceptor | 11/354 | 2.62E-05 | 0.001534 | 0.001339 | 11 |
| MF | GO:0004866 | endopeptidase inhibitor activity | 13/354 | 3.19E-05 | 0.001699 | 0.001483 | 13 |
| MF | GO:0030414 | peptidase inhibitor activity | 13/354 | 4.83E-05 | 0.002355 | 0.002055 | 13 |
| MF | GO:0050661 | NADP binding | 7/354 | 6.69E-05 | 0.003011 | 0.002628 | 7 |
| MF | GO:0016712 | oxidoreductase activity, acting on paired donors, with incorporation or reduction of molecular oxygen, reduced flavin or flavoprotein as one donor, and incorporation of one atom of oxygen | 6/354 | 9.36E-05 | 0.003912 | 0.003414 | 6 |
| MF | GO:0061134 | peptidase regulator activity | 14/354 | 0.000158 | 0.00615 | 0.005367 | 14 |
| MF | GO:0005506 | iron ion binding | 11/354 | 0.00018 | 0.006564 | 0.005728 | 11 |
| MF | GO:0005125 | cytokine activity | 14/354 | 0.000197 | 0.006786 | 0.005922 | 14 |
| MF | GO:0004857 | enzyme inhibitor activity | 18/354 | 0.000225 | 0.007298 | 0.006369 | 18 |
| MF | GO:0001664 | G protein-coupled receptor binding | 15/354 | 0.000517 | 0.015904 | 0.013879 | 15 |
| MF | GO:0050840 | extracellular matrix binding | 6/354 | 0.000642 | 0.018788 | 0.016396 | 6 |
| MF | GO:0008106 | alcohol dehydrogenase (NADP+) activity | 4/354 | 0.000899 | 0.025031 | 0.021845 | 4 |
| MF | GO:0016209 | antioxidant activity | 7/354 | 0.001025 | 0.026549 | 0.023169 | 7 |
| MF | GO:0030283 | testosterone dehydrogenase [NAD(P)] activity | 3/354 | 0.001044 | 0.026549 | 0.023169 | 3 |
| MF | GO:0005201 | extracellular matrix structural constituent | 10/354 | 0.00152 | 0.037058 | 0.03234 | 10 |
| MF | GO:0045028 | G protein-coupled purinergic nucleotide receptor activity | 3/354 | 0.001758 | 0.041142 | 0.035904 | 3 |
| MF | GO:0044594 | 17-beta-hydroxysteroid dehydrogenase (NAD+) activity | 3/354 | 0.002206 | 0.048585 | 0.0424 | 3 |
| MF | GO:0016903 | oxidoreductase activity, acting on the aldehyde or oxo group of donors | 5/354 | 0.002242 | 0.048585 | 0.0424 | 5 |
