# Supplementary material for: Analysis of immune characteristics and inflammatory mechanisms in COPD patients: a multi-layered study combining bulk and single-cell transcriptome analysis and machine learning
Source: Front Med (Lausanne). 2025 Jul 21;12:1592802. doi: 10.3389/fmed.2025.1592802 (PMC12318759; doi:10.3389/fmed.2025.1592802)
Supplement: Supplementary file 3 [file Table_3.docx]

**Supplementary Table 3. KEGG Pathway Enrichment Analysis Results of Differentially Expressed Genes**

| **Category** | **Term** | **Count** | **percent** | **P_Value** | **Benjamini** |
| --- | --- | --- | --- | --- | --- |
| KEGG_PATHWAY | Metabolic pathways | 55 | 12.9 | 1.60E-05 | 2.90E-03 |
| KEGG_PATHWAY | Metabolism of xenobiotics by cytochrome P450 | 10 | 2.3 | 2.50E-05 | 2.90E-03 |
| KEGG_PATHWAY | Steroid hormone biosynthesis | 9 | 2.1 | 3.20E-05 | 2.90E-03 |
| KEGG_PATHWAY | Arachidonic acid metabolism | 7 | 1.6 | 1.40E-03 | 9.60E-02 |
| KEGG_PATHWAY | Chemical carcinogenesis - DNA adducts | 7 | 1.6 | 2.80E-03 | 1.60E-01 |
| KEGG_PATHWAY | Ovarian steroidogenesis | 6 | 1.4 | 3.50E-03 | 1.60E-01 |
| KEGG_PATHWAY | Thyroid hormone synthesis | 7 | 1.6 | 4.00E-03 | 1.60E-01 |
| KEGG_PATHWAY | Type I diabetes mellitus | 5 | 1.2 | 1.10E-02 | 3.30E-01 |
| KEGG_PATHWAY | Rheumatoid arthritis | 7 | 1.6 | 1.10E-02 | 3.30E-01 |
| KEGG_PATHWAY | Retinol metabolism | 6 | 1.4 | 1.20E-02 | 3.30E-01 |
| KEGG_PATHWAY | Staphylococcus aureus infection | 7 | 1.6 | 1.30E-02 | 3.30E-01 |
| KEGG_PATHWAY | Chemical carcinogenesis - reactive oxygen species | 11 | 2.6 | 1.50E-02 | 3.30E-01 |
| KEGG_PATHWAY | Viral protein interaction with cytokine and cytokine receptor | 7 | 1.6 | 1.60E-02 | 3.30E-01 |
| KEGG_PATHWAY | Parathyroid hormone synthesis, secretion and action | 7 | 1.6 | 2.00E-02 | 4.00E-01 |
| KEGG_PATHWAY | TNF signaling pathway | 7 | 1.6 | 2.80E-02 | 5.10E-01 |
| KEGG_PATHWAY | Mucin type O-glycan biosynthesis | 4 | 0.9 | 3.60E-02 | 6.00E-01 |
| KEGG_PATHWAY | Alanine, aspartate and glutamate metabolism | 4 | 0.9 | 3.80E-02 | 6.00E-01 |
| KEGG_PATHWAY | Cell adhesion molecules | 8 | 1.9 | 4.00E-02 | 6.00E-01 |
| KEGG_PATHWAY | IL-17 signaling pathway | 6 | 1.4 | 4.10E-02 | 6.00E-01 |
