# Supplementary material for: Analysis of immune characteristics and inflammatory mechanisms in COPD patients: a multi-layered study combining bulk and single-cell transcriptome analysis and machine learning
Source: Front Med (Lausanne). 2025 Jul 21;12:1592802. doi: 10.3389/fmed.2025.1592802 (PMC12318759; doi:10.3389/fmed.2025.1592802)
Supplement: Supplementary file 4 [file Table_4.docx]

**Supplementary Table 4. GSEA Results for COPD Samples and Healthy Control Samples.**

| **Description** | **setSize** | **enrichmentScore** | **NES** | **pvalue** | **p.adjust** | **qvalue** |
| --- | --- | --- | --- | --- | --- | --- |
| HALLMARK_INFLAMMATORY_RESPONSE | 198 | 5.29E-01 | 2.30E+00 | 1.00E-10 | 5.00E-09 | 2.95E-09 |
| HALLMARK_TNFA_SIGNALING_VIA_NFKB | 194 | 4.41E-01 | 1.91E+00 | 5.75E-07 | 1.35E-05 | 7.97E-06 |
| HALLMARK_KRAS_SIGNALING_UP | 192 | 4.39E-01 | 1.91E+00 | 8.11E-07 | 1.35E-05 | 7.97E-06 |
| HALLMARK_MTORC1_SIGNALING | 189 | 4.04E-01 | 1.75E+00 | 1.84E-05 | 2.30E-04 | 1.36E-04 |
| HALLMARK_ALLOGRAFT_REJECTION | 193 | 4.01E-01 | 1.74E+00 | 4.03E-05 | 4.03E-04 | 2.38E-04 |
| HALLMARK_COMPLEMENT | 194 | 3.79E-01 | 1.64E+00 | 1.47E-04 | 1.23E-03 | 7.22E-04 |
| HALLMARK_EPITHELIAL_MESENCHYMAL_TRANSITION | 195 | 3.78E-01 | 1.64E+00 | 2.66E-04 | 1.90E-03 | 1.12E-03 |
| HALLMARK_IL2_STAT5_SIGNALING | 193 | 3.74E-01 | 1.62E+00 | 4.45E-04 | 2.47E-03 | 1.46E-03 |
| HALLMARK_INTERFERON_GAMMA_RESPONSE | 194 | 3.66E-01 | 1.58E+00 | 3.99E-04 | 2.47E-03 | 1.46E-03 |
| HALLMARK_XENOBIOTIC_METABOLISM | 192 | 3.57E-01 | 1.55E+00 | 1.88E-03 | 8.55E-03 | 5.04E-03 |
| HALLMARK_GLYCOLYSIS | 193 | 3.56E-01 | 1.55E+00 | 1.83E-03 | 8.55E-03 | 5.04E-03 |
| HALLMARK_REACTIVE_OXYGEN_SPECIES_PATHWAY | 47 | 5.07E-01 | 1.75E+00 | 2.53E-03 | 9.82E-03 | 5.79E-03 |
| HALLMARK_ESTROGEN_RESPONSE_LATE | 196 | 3.40E-01 | 1.48E+00 | 2.55E-03 | 9.82E-03 | 5.79E-03 |
| HALLMARK_ESTROGEN_RESPONSE_EARLY | 193 | 3.48E-01 | 1.51E+00 | 3.20E-03 | 1.14E-02 | 6.73E-03 |
| HALLMARK_NOTCH_SIGNALING | 31 | -5.24E-01 | -1.71E+00 | 6.12E-03 | 2.04E-02 | 1.20E-02 |
| HALLMARK_HEME_METABOLISM | 189 | -3.10E-01 | -1.38E+00 | 7.62E-03 | 2.38E-02 | 1.40E-02 |
| HALLMARK_IL6_JAK_STAT3_SIGNALING | 87 | 3.99E-01 | 1.53E+00 | 1.09E-02 | 3.19E-02 | 1.88E-02 |
| HALLMARK_FATTY_ACID_METABOLISM | 152 | 3.37E-01 | 1.42E+00 | 1.37E-02 | 3.81E-02 | 2.25E-02 |
| HALLMARK_CHOLESTEROL_HOMEOSTASIS | 71 | -3.81E-01 | -1.47E+00 | 1.52E-02 | 4.00E-02 | 2.36E-02 |
| HALLMARK_APOPTOSIS | 159 | 3.16E-01 | 1.34E+00 | 1.89E-02 | 4.73E-02 | 2.79E-02 |
| HALLMARK_UV_RESPONSE_DN | 135 | -3.16E-01 | -1.34E+00 | 2.00E-02 | 4.76E-02 | 2.80E-02 |
